# A review on regional convection‐permitting climate modeling: Demonstrations, prospects, and challenges

**DOI:** 10.1002/2014RG000475

**Published:** 2015-05-27

**Authors:** Andreas F. Prein, Wolfgang Langhans, Giorgia Fosser, Andrew Ferrone, Nikolina Ban, Klaus Goergen, Michael Keller, Merja Tölle, Oliver Gutjahr, Frauke Feser, Erwan Brisson, Stefan Kollet, Juerg Schmidli, Nicole P. M. van Lipzig, Ruby Leung

**Affiliations:** ^1^ National Center for Atmospheric ResearchBoulderColoradoUSA; ^2^ Wegener Center for Global and Climate Change (WEGC)University of GrazGrazAustria; ^3^ Earth Sciences DivisionLawrence Berkeley National LaboratoryBerkeleyCaliforniaUSA; ^4^ Météo‐France/CNRSCNRM‐GAMEToulouseFrance; ^5^ Luxembourg Institute of Science and Technology, Environmental Research and Innovation DepartmentEnvironmental Resource CenterBelvauxLuxembourg; ^6^ Institute for Atmospheric and Climate ScienceETH ZurichZurichSwitzerland; ^7^ Meteorological InstituteUniversity of BonnBonnGermany; ^8^ Jülich Supercomputing CentreResearch Centre JülichJülichGermany; ^9^ Centre for High‐Performance Scientific Computing in Terrestrial SystemsABC/J GeoverbundJülichGermany; ^10^ Center for Climate Systems ModelingETH ZurichZurichSwitzerland; ^11^ Institute of GeographyJustus‐Liebig Universität GießenGiessenGermany; ^12^ Regional and Environmental Sciences, Department of Environmental MeteorologyUniversity of TrierTrierGermany; ^13^ Institute for Coastal ResearchHelmholtz‐Zentrum Geesthacht Centre for Materials and Coastal ResearchGeesthachtGermany; ^14^ Institut für Atmosphäre und UmweltGoethe‐Universitt Frankfurt am MainFrankfurtGermany; ^15^ Agrosphere (IBG‐3)Research Centre JülichJülichGermany; ^16^ Department of Earth and Environmental SciencesKU LeuvenLeuvenBelgium; ^17^ Pacific Northwest National LaboratoryRichlandWashingtonUSA

**Keywords:** convection‐permitting modeling, added value, climate, cloud resolving, nonhydrostatic modeling, high resolution

## Abstract

Regional climate modeling using convection‐permitting models (CPMs; horizontal grid spacing <4 km) emerges as a promising framework to provide more reliable climate information on regional to local scales compared to traditionally used large‐scale models (LSMs; horizontal grid spacing >10 km). CPMs no longer rely on convection parameterization schemes, which had been identified as a major source of errors and uncertainties in LSMs. Moreover, CPMs allow for a more accurate representation of surface and orography fields. The drawback of CPMs is the high demand on computational resources. For this reason, first CPM climate simulations only appeared a decade ago. In this study, we aim to provide a common basis for CPM climate simulations by giving a holistic review of the topic. The most important components in CPMs such as physical parameterizations and dynamical formulations are discussed critically. An overview of weaknesses and an outlook on required future developments is provided. Most importantly, this review presents the consolidated outcome of studies that addressed the added value of CPM climate simulations compared to LSMs. Improvements are evident mostly for climate statistics related to deep convection, mountainous regions, or extreme events. The climate change signals of CPM simulations suggest an increase in flash floods, changes in hail storm characteristics, and reductions in the snowpack over mountains. In conclusion, CPMs are a very promising tool for future climate research. However, coordinated modeling programs are crucially needed to advance parameterizations of unresolved physics and to assess the full potential of CPMs.

## Key Points

- Convection‐permitting climate models reduce errors in large‐scale models
- Added value in convective processes, regional extremes, and over mountains
- Discusses challenges/potentials of convection‐permitting climate simulations

## 1 Introduction

A fundamental challenge in climate science is the scale gap between climate information provided by climate models and the needs of impact researchers, stakeholders, and policy makers. Although climate mitigation and adaptation measures are evaluated and applied at local to regional level, current state‐of‐the‐art large‐scale models (LSM) (global climate model (GCM) and regional climate model (RCM)) operate at horizontal grid spacing (Δ*x*) larger than 100 km [e.g., *Intergovernmental Panel on Climate Change (IPCC)*, 2013; *Taylor et al.*, 2012] and *δ* *x* = 10 km [e.g., *Jacob et al.*, 2014], respectively.

In addition to the mismatch between actionable and provided spatial information, important processes that are not resolved with grid spacings of climate models must be parameterized. These processes strongly affect regional and global climate, and their parameterizations are considered as a major source for model errors and uncertainty in future climate projections [e.g., *Ellingson et al.*, 1991; *Henderson‐Sellers et al.*, 1993; *Pedersen and Winther*, 2005; *Déqué et al.*, 2007].

One particularly critical subgrid process in climate models is the representation of deep convection through convection parameterization schemes. Deep convection is a dominant source of precipitation in many regions of the world and contributes disproportionally to extreme events such as flash floods and landslides through heavy precipitation associated with mesoscale convective systems, squall lines, and tropical cyclones [e.g., *Slingo et al.*, 1994; *Arakawa*, 2004]. Moreover, upward transport and detrainment of condensate and aerosols into the upper troposphere by deep convection is also critical to the climate system because of the resulting vertical mixing of the troposphere and the radiative forcing of upper level clouds. Parameterizing deep convection is challenging because the triggering of deep convection emerges from an interplay of processes acting at scales from the microscale to the synoptic scale. Besides the triggering of deep convective updrafts, the assumptions made for the entrainment and detrainment of convective plumes (see *de Rooy et al.* [2013] for a review) and for their precipitation efficiency [e.g., *Renno et al.*, 1994; *Emanuel*, 1991] remain rather crude. However, climate projections with GCM and RCM show that the parameterization of these characteristics of deep convective clouds make up for the largest uncertainties of projected large‐scale parameters such as the climate sensitivity [*Knight et al.*, 2007; *Sanderson et al.*, 2008; *Sherwood et al.*, 2014]. In addition, convection parameterization schemes interact with many other parameterization schemes, such as microphysics, radiation, and planetary boundary layer schemes, such that weaknesses in convection parameterization schemes can imply far‐reaching consequences through nonlinearities. The inherent assumptions made in convection parameterization schemes were assessed critically in previous studies [e.g., *Molinari and Dudek*, 1992; *Romps*, 2010; *Jones and Randall*, 2011; *Arakawa et al.*, 2011]. The use of convection parameterization schemes leads to common errors such as misrepresentation of the diurnal cycle of convective precipitation [e.g., *Dai et al.*, 1999; *Brockhaus et al.*, 2008], underestimation of dry days and overestimation of low‐precipitation event frequency [e.g., *Berg et al.*, 2013], and the underestimation of hourly precipitation intensities [e.g., *Prein et al.*, 2013a; *Fosser et al.*, 2014; *Ban et al.*, 2014] and is largely responsible for several tropical biases associated with intraseasonal to interannual variability [*Song and Zhang*, 2009; *Zhang and Song*, 2010; *Chikira*, 2010]. It is important to mention that recently developed parameterization schemes lead to improvements of several of these common errors including the simulation of precipitation intensities [*Donner et al.*, 2011], intraseasonal variability [*Benedict et al.*, 2013], and diurnal cycles [*Bechtold et al.*, 2014].

A promising remedy to the error‐prone climate simulations using convective parameterizations is the use of convection‐permitting model (CPM) (horizontal grid spacing <4 km) that operates on the kilometer scale. Several different terms have been used for CPMs throughout the literature. Terminologies such as cloud‐resolving, convection‐resolving, cloud‐permitting, or convection‐permitting simulations have been frequently used interchangeably [e.g., *Satoh et al.*, 2008; *Prein et al.*, 2013a; *Ban et al.*, 2014]. We decided to use the term “convection‐permitting simulations” because “cloud‐resolving” or “convection‐resolving” simulations could be misleading since clouds contain energy on scales as small as the Kolmogorov scale (the smallest scales at which kinetic energy is converted into heat), which we are not intending to resolve for climate simulations.

In mesoscale atmospheric research, CPMs have been used for decades in studies of idealized cloud systems and real weather events. Since the beginning of the 21st century, advances in high‐performance computing allowed steady refinement of the numerical grids of climate models well beyond 10 km. At these scales, convection parameterization schemes may eventually be switched off as deep convection starts to be resolved explicitly [e.g., *Weisman et al.*, 1997]. Section 3 reviews the minimum grid spacing required for switching off the convection parameterization and of experiences collected from simulations with even smaller grid spacings. Besides explicitly resolving deep convection, CPMs also offer the advantage of improving the representation of fine‐scale orography and variations of surface fields. This can be especially beneficial in mountainous regions and in areas with heterogeneous land surfaces (e.g., coastal and urban regions, wetlands, and patchy land covers) [e.g., *Prein et al.*, 2013a, 2013b; *Lauwaet et al.*, 2012; *Trusilova et al.*, 2013]. Resolving fine‐scale surface heterogeneity is also advantageous because it is an important forcing for deep convection.

**Figure 1:**
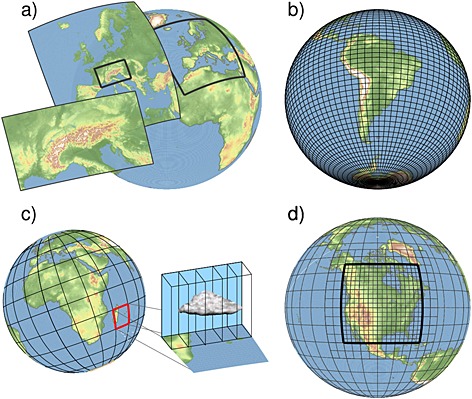
Visualization of four different modeling approaches for convection‐permitting climate simulations: (a) limited‐area modeling, (b) global CPM climate simulations, (c) superparameterizations, and (d) stretched‐grid models.

Four basic modeling approaches used to perform CPM climate simulations are visualized in Figure 1. The first approach, limited‐area modeling, is used most frequently (Figure 1a). This approach telescopically nests limited‐area domains at decreasing horizontal grid spacings with boundary conditions provided by a GCM or reanalysis until convection‐permitting scales are reached. This approach was first used in numerical weather prediction, and numerous studies demonstrated the benefits of CPM climate simulations forecasts of severe weather [e.g., *Bernardet et al.*, 2000; *Done et al.*, 2004; *Schwartz et al.*, 2009; *Weusthoff et al.*, 2010] or for simulating rainfall intensity spectra [*Davis et al.*, 2006]. Pioneering work toward CPM climate simulations was provided by [*Grell et al.*, 2000] who performed 14 month long simulations with Δ*x* = 1 km and showed drastic changes in the seasonal average precipitation patterns compared to LSM simulations. More recent studies have performed CPM simulations on time scales longer than 1 year to investigate climatological features in CPM simulations [e.g., *Brisson et al.*, 2015; *Chan et al.*, 2013; *Ban et al.*, 2014; *Fosser et al.*, 2014; *Kendon et al.*, 2014; *Junk et al.*, 2014; *Tölle et al.*, 2014; *Prein et al.*, 2013b; *Rasmussen et al.*, 2011; *Ikeda et al.*, 2010; *Gensini and Mote*, 2014; *Chan et al.*, 2013; *Kendon et al.*, 2012; *Knote et al.*, 2010; *Rasmussen et al.*, 2014].

The second approach is to run global integrations at convection‐permitting grid spacing (Figure 1b). This approach is most rigorous as it allows for a seamless simulation of processes ranging from the scale of convective clouds to the global scale [e.g., *Miura et al.*, 2007; *Satoh et al.*, 2008; *Putman and Suarez*, 2011; *Miyamoto et al.*, 2013]. At the moment, the large computational costs of this technique limited the runtime of such simulations to a few days or 1 month. Based on the good match between predicted [*Moore*, 1965] and realized availability of computational resources, long‐term simulations with global CPMs may be feasible in one to two decades.

To reduce the computational challenges of global CPMs, the third approach uses GCM with the so‐called superparameterizations (Figure 1c) [*Grabowski and Smolarkiewicz*, 1999; *Khairoutdinov and Randall*, 2001]. Within this framework, each GCM grid column embeds a two‐dimensional cloud‐resolving model. This framework has proven useful as it leads to improvements in simulating both extremely light and extremely intense precipitations [*Li et al.*, 2012], the Madden–Julian oscillation [*Benedict and Randall*, 2009], propagating organized convective systems [*Pritchard et al.*, 2011; *Kooperman et al.*, 2013], the Asian monsoon [*Goswami et al.*, 2013], and the El Niño–Southern Oscillation [*Stan et al.*, 2010]. *Randall et al.* [2003] estimated that the computational costs of using superparameterizations are factor of 10^2^ to 10^3^ larger than the costs for traditional large‐scale model (LSM). However, this technique is still computationally inexpensive compared to the second approach using global CPMs, which is 10^6^ times more expensive than traditional LSM [*Randall et al.*, 2003].

The fourth approach uses stretched‐grid models [e.g., *Schmidt*, 1977; *Staniforth and Mitchell*, 1978] or variable resolution models [e.g., *Skamarock et al.*, 2012; *Rauscher et al.*, 2013; *Hagos et al.*, 2013] to reduce the regional grid spacing over areas of interest, while larger grid spacing is used elsewhere for computational efficiency (Figure 1d). The UK Met Office, for example, uses this approach for their operational weather forecast [*Tang et al.*, 2013] in which the global domain at Δ*x* = 17 km is refined to Δ*x* = 4 km over Europe and to Δ*x* = 1.4 km over the British Isles. Using this stretched‐grid approach, *Tang et al.* [2013] showed that results obtained for an ensemble of convective cases compare well with results obtained using the first approach of grid telescoping. The Model for Prediction Across Scales [*Skamarock et al.*, 2012] and the Icosahedral nonhydrostatic general circulation model [*Wan et al.*, 2013] are other examples of global variable resolution models with nonhydrostatic dynamics and potential for convection‐permitting modeling. Multiscale models using adaptive mesh refinement (i.e., dynamic local refinement of the grid) might also have considerable potential to improve the representation of deep convection at a reasonable cost [*Slingo et al.*, 2009]. As an example, adaptive meshes have already been used in the climate community to successfully simulate the retreat of continental‐scale ice sheets [*Cornford et al.*, 2013].

Only the first approach, grid telescoping, is considered in this review, and various forms of this methods will be studied and compared in section 4.

Since the beginning of the 21st century more and more studies have focused on CPM climate simulations and there is now a strong need to synthesize these studies and to build the foundation and common basis for future advances in climate modeling. Furthermore, impact researchers and stakeholders should be informed of what to expect from CPM climate simulations. This review paper aims to provide this kind of scientific basis by summarizing the knowledge acquired up to now and by highlighting existing challenges and important research questions in this field. In particular, we review the following: (1) What grid spacing is needed for CPM climate simulations (section 3)? (2) What is the best downscaling strategy to convection‐permitting scales (section 4)? (3) What are the most important model components that require further development (section 5)? (4) What are, in theory, the added values of CPM climate simulations compared to LSM simulations (section 6.1)? (5) What added values could actually be demonstrated in practical applications (section 6)? (6) And what can we learn from CPM about future climate change that is not assessable from LSM (section 7)?

## 2 A Summary of CPM Climate Simulations Reviewed

**Figure 2:**
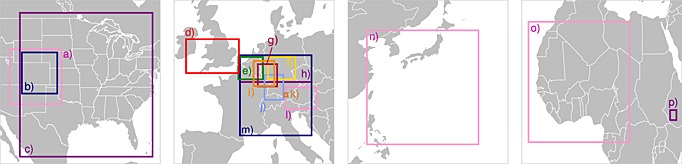
(a–p) Summary of the domains (schematic) used in the different CPM climate simulations that are addressed in this paper. The acronyms are defined in Table 1.

In this section, we provide a brief summary of the most important CPM climate simulations reviewed in this paper. Their selection is based on a careful literature review. Figure 2 shows an overview of the domains of CPMs investigated in individual studies. Most studies focus on Europe with domains located over the Alps, Germany, Belgium, and the UK. Three studies from the U.S. are included as well: two focusing on Colorado and one on the U.S. east of the Continental Divide. Two studies investigate African climates focusing on the Kilimanjaro and the Sahel regions, and one CPM climate simulation from Asia is also included which focuses on the northwestern Pacific Ocean. In Figure 2 we see a large variety of domain sizes starting from about 50 km × 50 km (Figure 2k) [*Grell et al.*, 2000] to about 2000 km × 3000 km (Figure 2c) [*Gensini and Mote*, 2014]. The reviewed studies also differ in the downscaling strategy, and the simulation periods range from relatively short case studies to 30 years (see Table 1).

**Table 1:** Overview of the Investigated Convection‐Permitting Model (CPM) Climate Simulations

Domain (Figure 2)	Model	Downscaling Strategy	Period (Unless Stated Otherwise All Simulations Start on 1 January and End on 31 December of the Indicated Years)^f^	Publication
a	WRF^a^	NARR 36 km → WRF 4 km	2001–2008	*Prein et al.* [2013b]
a	WRF^a^	NARR 36 km → WRF 4 km	01.11–01.05 for 2001–2008	*Rasmussen et al.* [2011]; *Ikeda et al.* [2010]; *Liu et al.* [2011]
a	WRF^a^	NARR 36 km → WRF 4 km; NARR perturbed by CCSM3 36 km → WRF 4 km	2001–2008 and 2042–2049 (A1B)	*Rasmussen et al.* [2014]
b	WRF^a^	GFDL‐CM2.1 → GFDL‐AM2.1 50 km → WRF 1.3 km	10 largest extreme events during June, July, and August (JJA) in 1971–2000 and 2041–2070 (A2)	*Mahoney et al.* [2012]
b	WRF^a^	GFDL‐AM2.1/WRF/CGCM3 50 km → WRF 4 km → WRF 1.3 km	10 strongest extreme events during June, July, and August (JJA) in 1971–2000 and 2041–2070 (A2)	*Mahoney et al.* [2013]
c	WRF^a^	CCSM3 150 km → WRF 4 km	01.03–31.05 for 1980–1990	*Gensini and Mote* [2014]
d	Modified UKV^b^	ERA‐Interim → 12 km → 1.5 km	1989–2008	*Chan et al.*, 2013 [2014b, 2013]; *Kendon et al.* [2012]
d	Modified UKV^b^	HadGEM3‐RA 60 km → 12 km → 1.5 km	1996–2009 and 2087–2099 (RCP8.5)	*Kendon et al.* [2014]
e	COSMO‐CLM^c^	ERA‐Interim 80 km → 25 km → 7 km → 3 km	2000–2010	*Brisson et al.* [2015]
f	COSMO‐CLM^c^	ERA40/European Centre/Hamburg (ECHAM5) → 18 km → 7 km → 1.3 km	1970–1975 and 2070–2075 (A1B)	*Tölle et al.* [2014]
g	COSMO‐CLM^c^	ECHAM5 120 km → 18 km → 5 km → 2.8 km	1960–1969 and 2015–2024 (A1B)	*Knote et al.* [2010]
h	COSMO‐CLM^c^	IFS 25 km → 25 km → 2.2 km	01.06.2006–30.06.2006	*Hohenegger et al.* [2008, 2009]
i	COSMO‐CLM^c^	ERA40/ECHAM5 → 18 km → 4.5 km → 1.3 km	1991–2000 (ERA40/C20) and 2041–2050 (A1B) and 2091–2100 (A1B)	*Junk et al.* [2014]
j	COSMO‐CLM^c^	ERA‐40 → 50 km → 7 km → 3 km	1968–1999	*Fosser et al.* [2014]
k	MM5^d^	ECHAM3 120 km → 60 km → 15 km → 5 km → 1 km	14 months	*Grell et al.* [2000]
l	COSMO‐CLM^c^; MM5^d^; WRF^a^	IFS 25 km → 10km → 3 km	June, July, and August (JJA) 2007 and December, January, and February (DJF) 2007/2008	*Prein et al.* [2013a]
m	COSMO‐CLM^c^	ERA‐Interim 80 km → 12 km → 2.2 km	1998–2007	*Ban et al.* [2014]
m	COSMO‐CLM^c^	MPI‐ESM‐LR 200 km → 12 km → 2.2 km	1991–2000 and 2081–2090 (RCP8.5)	*Ban et al.* [2015]
m	COSMO‐CLM^c^	ERA‐Interim 80 km → 12 km → 2.2 km	03.07.2007–13.07.2007	M. Keller et al. (submitted manuscript, 2015)
m	COSMO‐CLM^c^	IFS 25 km → 2.2 km/1.1 km	01.07.2006–31.07.2006	*Langhans et al.* [2012b, 2013]
m	COSMO‐CLM^c^	IFS 25 km → 4.4 km/2.2 km/1.1 km/0.55 km	01.07.2006–31.07.2006	*Langhans et al.* [2012a]
n	NHM2^e^	AGCM20 20 km → NHM2 2 km	Six most intense tropical cyclones from 1979 to 2003 and 2075 to 2099	*Kanada et al.* [2013]
o	MetUM^b^	IFS 25 km → 12 km → 4 km	25.07.2006–02.09.2006	*Taylor et al.* [2013]; *Pearson et al.* [2014]
p	WRF^a^	ERA‐Interim 80 km → 39 km → 13 km → 3.25 km → 0.8 km	01.08.2005–31.08.2005 and 01.04.2006–31.04.2006	*Mölg and Kaser* [2011]

a Weather Research and Forecasting Model [*Skamarock et al.*, 2005].

b One of the several configurations of the Met Office Unified Model [*Cullen*, 1993].

c COSMO model in Climate Mode [*Rockel et al.*, 2008; *Steppeler et al.*, 2003].

d Fifth‐Generation Mesoscale Model [*Dudhia*, 1993].

e Two kilometer mesh nonhydrostatic model [*Murakami et al.*, 2012].

f Dates are formatted as “day.month.year.”

Additionally, the parent‐grid ratios, i.e., the integer parent‐to‐nest ratios of the horizontal grid spacing, differ widely among individual experiments. Most studies use a parent‐grid ratio between 1:3 and 1:9, except one study that used a parent‐grid ratio of 1:38 [*Gensini and Mote*, 2014]. In the downscaling chain toward CPM simulations, many modelers avoided simulations in the so‐called “gray zone” (grid spacing between 10 km and 4 km) where some assumptions used in parameterizations of deep convection are violated and deep convection is insufficiently resolved to be modeled explicitly. Possible impacts of the domain size, the parent‐grid ratio, and the downscaling strategy are discussed in section 4.

## 3 What Grid Spacing Is Needed for CPM Climate Simulations?

The energy spectrum of deep convective clouds is continuous across kilometer scales without an apparent energetic gap indicating a scale separation [*Gage*, 1979; *Nastrom and Gage*, 1985; *Wyngaard*, 2004; *Moeng et al.*, 2010]. To resolve a larger portion of the flow, the steady increase in computing resources thus evokes a continued quest for finer grid spacings in both weather and climate simulations. However, since numerical resolution comes with additional costs, we need to ask ourselves which grid spacing is sufficient for CPM simulations. This section addresses this question with a focus on the horizontal grid spacing. The distribution of vertical levels is typically indifferent to the one of convection‐permitting models in weather models, and we refer the interested reader to textbooks on mesoscale modeling for further details [e.g., *Pielke*, 2013].

The upper bound on the horizontal grid spacing of convection‐permitting simulations was investigated by *Weisman et al.* [1997] using idealized squall line simulations. Their findings demonstrated the inability to represent accurately nonhydrostatic dynamics with horizontal grid spacings larger than 4 km. The convective mass flux was overestimated once this threshold was exceeded and resulted in “grid‐scale storms.” The latter emerge as convective instability is forced onto an unrealistically coarse scale, hence overestimating the convective mass flux and precipitation. Thus, CPM at 4 km or coarser grid spacings may not always yield improvements over LSMs. Applying convection parameterization schemes at such grid spacings may overcome the aforementioned issues of underresolved convection [*Deng and Stauffer*, 2006; *Lean et al.*, 2008; *Roberts and Lean*, 2008]. Nevertheless, some studies have reported improved model behavior at 4 km grid spacing without convection parameterization schemes [*Done et al.*, 2004; *Weisman et al.*, 2008; *Schwartz et al.*, 2009; *Prein et al.*, 2013b]. Given this uncertainty, it seems prudent to use horizontal grid spacings of less than 4 km for CPM climate simulations.

Indeed, the explicit treatment of convection using models with grid spacings less than 4 km has led to considerable improvements of quantitative precipitation forecasts [*Benoit et al.*, 2002; *Richard et al.*, 2007; *Lean et al.*, 2008; *Skamarock and Klemp*, 2008; *Schwartz et al.*, 2009; *Weusthoff et al.*, 2010; *Baldauf et al.*, 2011] and climate simulations [e.g., *Kendon et al.*, 2012; *Ban et al.*, 2014; *Fosser et al.*, 2014] and provided remedy to issues that plagued LSMs for too long. Particularly during the summer, the timing of the onset and peak of convective precipitation over land occurred too early in LSMs [*Dai et al.*, 1999; *Randall et al.*, 2003; *Guichard et al.*, 2004; *Bechtold et al.*, 2004; *Brockhaus et al.*, 2008; *Hohenegger et al.*, 2008]. These improvements are evident even though, as discussed in section 5.3, the assumptions made in the applied turbulence parameterizations break down at kilometer‐scale grid spacings [*Wyngaard*, 2004; *Moeng et al.*, 2010].

In the limit of extremely fine horizontal grid spacings, CPMs are thought to converge to large‐eddy simulations [*Bryan et al.*, 2003; *Cullen and Brown*, 2009; *Khairoutdinov et al.*, 2009]. The latter apply grid spacings on the order of 100 m to resolve the energy‐producing turbulent motion of the largest eddies within deep convective plumes. *Craig and Dörnbrack* [2008] found that the bulk cumulus growth as reflected in the evolution of the cloud's extent converges at a horizontal grid spacing of about 50 m. Other studies echo a similar requirement on the horizontal grid spacing in order to resolve the evolution and morphology of individual clouds or cloud systems [*Petch et al.*, 2002; *Adlerman and Droegemeier*, 2002; *Bryan et al.*, 2003; *Petch*, 2006; *Lang et al.*, 2007; *Fiori et al.*, 2010]. The conclusions in these studies were drawn based on the analyses of a variety of parameters such as maximum vertical velocity or profiles of vertical fluxes. However, we might ask ourselves if these are also appropriate criteria for our choice of horizontal grid spacings in CPM climate models.

It is here important to distinguish the purpose and concept of CPM climate simulations from that of weather forecasts or studies of individual cloud systems. At kilometer scales, deterministic predictability is limited to a few hours and small‐scale structures are dominated by stochastic processes [*Lorenz*, 1969; *Zhang et al.*, 2003; *Hohenegger and Schär*, 2007]. The goal of CPMs is to represent the overall statistics of an ensemble of convective elements (e.g., diurnal cycle of average rainfall and precipitation intensity) as well as the associated interactions with radiation, the Earth's surface, and the larger‐scale flow (e.g., the bulk latent heating). But what is an adequate horizontal grid spacing below 4 km that sufficiently resolves this interaction of deep convection with the climate system?

This question was addressed by *Schwartz et al.* [2009] who analyzed skill scores of quantities such as domain‐averaged precipitation from 4 km and 2 km simulations. Model results were analyzed at least 21 h after initialization, which is beyond the typical error doubling times of a few hours. Despite adding further details to the cloud structures and dynamics, the added scales seemed to be dominated by chaos without providing any added skill to the larger scales and mean precipitation. Their findings echo the previous findings by *Kain et al.* [2008]. Similar results were also obtained by *Langhans et al.* [2013] (see Table 1, domain m) from 18 day simulations of orographic convection at 2.2 km and 1.1 km. Their findings showed only minor differences in the mean diurnal cycle of cloud cover and precipitation.

**Figure 3:**
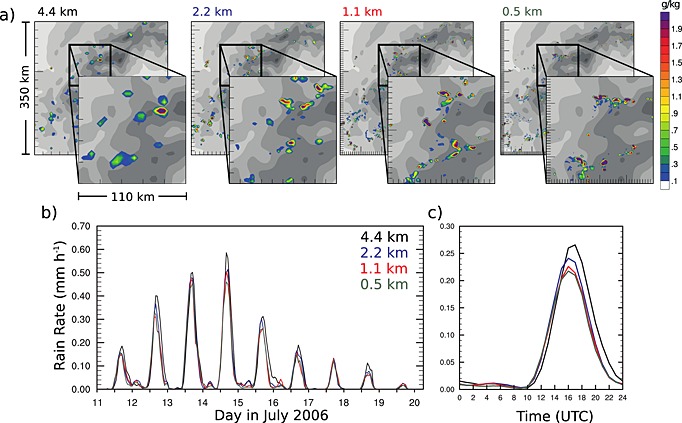
The behavior of a convection‐permitting simulation across kilometer‐scale grid spacings is illustrated for the example of orographic convection over the southwestern parts of the European Alps. (a) Snapshots of the cloud liquid water content of clouds within a 350 km × 350 km large subdomain of the full modeling domain. The increase in resolution decreases the size of individual clouds and increases the number of convective clouds. (b) The surface rain rate averaged over a larger domain covering the whole Alpine mountain range. (c) The 9 day average diurnal cycle obtained from the time series shown in Figure 3b. The magnitude and timing of surface precipitation are largely insensitive to the horizontal grid spacing (adapted from *Langhans et al.* [2012a], ©Copyright 2012 American Meteorological Society (AMS)).

The minimum requirement on the horizontal grid spacing to simulate the bulk heat and moisture tendencies and surface precipitation from an ensemble of convective cells has been addressed by *Langhans et al.* [2012a] (see Table 1, domain m). CPM climate simulations were performed over 9 days at horizontal grid spacing (Δ*x*) of 4.4 km, 2.2 km, 1.1 km, and 0.55 km. Figure 3a shows the simulated convective cloud liquid water content for a small subdomain and for the four different grid spacings. Although the convective clouds become smaller and smaller with decreasing grid spacing, little sensitivity is found in terms of mean surface precipitation (Figures 3b and 3c). The spread in the peak rain rate of the mean diurnal cylce (Figure 3c) between the 2.2 km simulation and the highest resolution run (0.5 km) amounts to about 15% and becomes even smaller for the 1.1 km simulation. The timing of the peak is also unaffected. Note again that the 4.4 km simulation deviates from the higher‐resolution runs by producing too much precipitation. This weak grid sensitivity is reflected also in the feedback to the large‐scale flow (as measured by the mentioned bulk tendencies) which appeared to converge across the investigated range of scales. Although their results might hold only for orographically triggered convection, their findings are particularly encouraging as it may not be necessary to resolve small‐scale turbulent eddies in order to simulate the area‐averaged precipitation. Equivalent studies are required over flat terrain to confirm this insensitivity for climate regimes less influenced by persistent forcing of boundary layer dynamics.

It is of interest to point out that simulations on even finer numerical grids will certainly benefit from better resolved topographic features (e.g., coast lines, lakes, and orography). Sufficient horizontal grid spacing is of particular importance to simulate the small‐scale variability of surface precipitation over complex terrain [*Colle and Mass*, 2000; *Zängl*, 2007; *Richard et al.*, 2007; *Langhans et al.*, 2011]. Note, however, that excellent verification results have been obtained for long‐term simulations of precipitation over the European Alps using 2 km grid spacing [*Hohenegger et al.*, 2008; *Langhans et al.*, 2013; *Ban et al.*, 2014; M. Keller et al., Evaluation of convection‐resolving models using satellite data: The diurnal cycle of summer convection over the Alps, submitted to *Meteorologische Zeitschrift*, 2015], indicating that such grid spacings are sufficient for simulations of rainfall even over highly complex terrain. Still, other sources of land surface heterogeneity (e.g., gradients in soil moisture, vegetation, and urban effects) might modulate locally the dynamics in the boundary layer [*Segal and Arritt*, 1992; *Taylor et al.*, 2011; *Froidevaux et al.*, 2014; *Lauwaet et al.*, 2010], and it remains unclear to what degree the induced secondary circulations affect precipitation statistics in climate simulations (and to what degree these effects could be successfully parameterized).

Certainly, the question emerges of how much deviation from the converged solution is tolerated in CPMs. Considering the weak grid sensitivity reported above from models running with grid spacings below 4 km and the fact that physical parameterizations result in a similar or even larger spread [*Wang et al.*, 2009; *Roh and Satoh*, 2014; M. Keller et al., submitted manuscript, 2015], it appears more urgent to address aspects of physical parameterizations first before further refining the horizontal grid spacing toward those applied in large‐eddy simulations. To prioritize the design of scale‐aware physical parameterizations seems promising for two reasons. First, our understanding of microphysical and turbulent processes at kilometer scales is poor (see also sections 5.2 and 5.3). More research is necessary in that direction even in order to design parameterizations for grids feasible nowadays. Second, large‐eddy simulations at regional and decadal scales will remain unaffordable for at least another decade or more such that parameterizations would constantly have to be adapted and retuned following a refinement of the grid. Another important open question is to what degree the statistical properties of interest are constrained and set by the small‐scale detail of individual clouds. For example, inaccuracies in the representation of the maximum vertical velocities in convective clouds (as reported by, e.g., *Varble et al.* [2014] or *Miyamoto et al.* [2013]) could obviously modulate the cloud cover and extreme precipitation statistics—both quantities we seek to project with CPM simulations. Even though the above mentioned studies are promising, more studies are thus required to attest a minor grid sensitivity of these climate‐relevant parameters across kilometer scales.

## 4 Downscaling Strategy

Dynamical downscaling is conceptually based on the generation of fine scales with RCM simulations initialized and driven by a coarse‐mesh GCM [*Dickinson et al.*, 1989; *Giorgi and Bates*, 1989]. Thereby, the fine scales are dynamically consistent with the large‐scale flow imposed as lateral boundary conditions [*Anthes et al.*, 1982]. Thus, RCMs can represent finer‐scale details compared to the driving GCMs and can reduce the degree of parameterized physics (e.g., deep convection in case of CPMs) and representation of surface heterogeneities (e.g., orography and coastlines).

In this section, we review the rationale behind specific downscaling strategies. Aspects addressed here include the nesting technique including the parent‐grid ratio, the effect of two‐way nesting, domain size, and nudging.

### 4.1 Impact of Nesting Strategy

How to downscale large‐scale GCM output to regional and local scales over a limited area is a common challenge of LSM and CPM. Usually, multiple nested limited‐area domains at decreasing horizontal grid spacings are applied until the convection‐permitting scale is reached. However, there is no common agreement on how many steps are needed and how small the parent‐grid ratio between the individual nests could be (i.e., see Table 1). Several studies found that the parent‐grid ratio should be larger than approximately 1:12 [e.g., *Antic et al.*, 2006; *Denis et al.*, 2003]. The ratio, however, strongly depends on the update frequency of the lateral boundary conditions, the investigated region, the model, and the domain size. *Brisson et al.* [2015] (Table 1, domain e) investigated the sensitivity of simulating precipitation with a CPM over Belgium by downscaling ERA‐Interim (Δ*x* = 80 km). They concluded that an intermediate nesting step with Δ*x* = 25 km was essential for the correct representation of precipitation in the CPM. Introducing an additional nest with Δ*x* = 7 km did not improve the results while both directly nesting the CPM into ERA‐Interim and replacing the Δ*x* = 25 km with the Δ*x* = 7 km nest lead to a strong dry bias. The deterioration in the ERA‐Interim to CPM nesting is probably related to the small parent‐grid ratio of 1:30 and the small domain size, while the deterioration in the intermediate Δ*x* = 7 km experiment is probably related to its grid spacing that is in the gray zone where assumptions of convection parameterizations starts to break down.

A method that can reduce disturbances at the lateral boundary conditions and allows for feedback from the nested model to its driving model is the so‐called two‐way nesting. In two‐way nesting, the coarse and the finer resolution simulations are run simultaneously. The coarser simulation provides boundary values for the finer, and the finer feeds its calculation back to the coarser domain [e.g., *Wang et al.*, 2014]. Additionally, the update frequency of the lateral boundary conditions of the finer nest is typically much higher (the time step of the coarse resolution run) compared to one‐way nesting experiments (typically one to several hours). Most of the here reviewed studies used a one‐way nesting strategy without any feedback from the fine to the coarse model. *Prein et al.* [2013a] (see Table 1, domain l) showed that there are no major differences between a one‐way and two‐way nested Fifth‐Generation Mesoscale Model (MM5) CPM climate simulation in the Eastern Alps.

Common guidelines that should be considered when planning a nesting strategy can be adopted from experiences with LSM [e.g., *Rummukainen*, 2010] or weather prediction models [*Warner et al.*, 1997]. Those include the orientation of the domain according to the large‐scale flow, avoiding boundaries that cut through mountains, or including source regions of phenomena in the CPM domain that are important for the study (e.g., mountain ranges).

### 4.2 Impact of Domain Size

Because of the high computational costs of CPM climate simulations, the domain size has to be chosen carefully. Results from LSM climate simulations showed that the quality of RCM simulations depends on the domain size [e.g., *Seth and Giorgi*, 1998; *Chomé et al.*, 2002; *Vannitsem and Chomé*, 2005]. Certainly, CPMs are indifferent in that respect. Small domains degrade the representation of large‐scale features [*de Ela et al.*, 2002] and large‐scale variability [*Vannitsem and Chomé*, 2005]; however, large domains can lead to strong deviations of the RCM simulation from its lateral boundary conditions and thus lead to undesired effects at the outflow boundary [*Vannitsem and Chomé*, 2005; *Jones et al.*, 1995; *Leduc and Laprise*, 2009; *von Storch*, 2005]. To prevent the occurrence of large‐scale differences, large‐scale nudging can be applied. Strings attached to this method are briefly discussed in section 4.3.

Regional climate models (RCMs) and CPMs need some spatial spin‐up (distance from the lateral boundaries to the point where the fine‐scale structures are reached) for the generation of fine‐scale features because they are forced by lateral boundary conditions which are given on a coarser scale and which are often physically inconsistent with the RCMs physics (e.g., different Reynolds numbers of the flow or different cloud liquid water contents). The extent of this spatial spin‐up depends on the speed of the flow, the parent‐grid ratio, hydrodynamic instabilities, nonlinear processes, and surface processes [*Laprise*, 2008]. It is hard to say how far away from the boundaries the fine‐scale features have reached their equilibrium. However, care has to be taken if small, computationally cheap, domains are used (*Jones et al.*'s, [1995] estimate was approximately 50 by 50 grid points for Δ*x* = 50 km) because they might be too small to allow the RCM to spin‐up. To provide a smoother transition between the lateral boundary conditions and the RCM simulation, a boundary relaxation zone with typically exponentially decreasing weights in the outermost ∼10 grid cells is applied [*Davies*, 1976; *Marbaix et al.*, 2003]. The width of the relaxation zone may vary according to the domain size, area, and parent‐grid ratio.

*Brisson et al.* [2015] (Table 1, domain e) investigated the influence of CPM domain size on simulated precipitation. They found that their two largest domains (180 × 180 and 200 × 200) show similar results, while the simulation quality deteriorated for smaller domains. They concluded that a spatial spin‐up of at least 40 grid cells is necessary for simulating realistic precipitation patterns in their Δ*x* = 3 km simulation. This was partly related to the morphogenesis of hydrometeors (e.g., graupel: heavily rimed snow particles) which were not considered in the driving model.

To account for the spatial spin‐up and possible lateral boundary reflection and noise, the nested model domain should be extended such that its boundaries are as far from the region of interest as possible [*Warner et al.*, 1997]. However, in the end the choice is largely constrained by computational costs—especially for CPM simulations.

### 4.3 Impact of Spectral Nudging

Spectral nudging [*von Storch et al.*, 2000; *Waldron et al.*, 1996] is a technique commonly used to constrain the large atmospheric conditions in RCM hindcast simulations to those of the driving model. Without spectral nudging regional climate models (RCMs) may yield large‐scale patterns that diverge from observations [e.g., *Castro et al.*, 2005; *Kanamitsu et al.*, 2010; *von Storch et al.*, 2000]. So far, spectral nudging has mainly been used for LSM hindcast simulations [*Feser et al.*, 2011], where added value was found for many meteorological variables such as near‐surface temperature or precipitation [e.g., *Feser*, 2006; *Meinke et al.*, 2006] and for phenomena such as tropical cyclones [*Feser and Barcikowska*, 2012; *Feser and von Storch*, 2008a, 2008b]. F. Feser and B. Schaaf (personal communication, 2014) tested the application of spectral nudging in a CPM hindcast simulation. They found very little differences between CPM runs with and without spectral nudging, probably related to their small domain size. For large‐domain CPM hindcast simulations, improvements of spectral nudging are likely to be similar than those found in LSM simulations.

For non‐hindcast driven climate model simulations the effect of spectral nudging still needs to be determined.

## 5 Important Components for CPM Climate Simulations

In this section, we investigate what numerical and physical model components are critical to improve CPM climate simulations and how to reach their full potential.

### 5.1 Numerical Formulation

Approximations made in LSMs are no longer valid at kilometer scales. The most prominent example is the hydrostatic approximation that simplifies the vertical equation of motion and assumes that the absolute vertical acceleration in the atmosphere is negligible. This is true for synoptic‐scale motions but breaks down for length scales less than 10 km [e.g., *Dutton*, 2002; *Holton and Hakim*, 2013]. Two sets of the nonhydrostatic governing equations have been used in convection‐permitting models so far. The fully compressible equations are most common as they are applicable from the global to the mesoscale without any approximations. The other used set is the soundproof approximation (proposed in various forms) which filters acoustic modes. Even though this equation set has been applied successfully in many small‐scale applications [*Prusa et al.*, 2008; *Duarte et al.*, 2015; *Klein et al.*, 2010], it has been argued that the validity of this approximation affects negatively the simulation of Rossby modes at larger horizontal scales [*Davies et al.*, 2003]. The drawback of the fully compressible set is that their solution include meteorologically unimportant sound waves. Due to their fast phase speed the time steps of fully compressible models must be excessively small in order to guarantee numerical stability. The numerical efficiency is, however, significantly improved by applying a time‐splitting scheme [e.g., *Klemp and Wilhelmson*, 1978; *Wicker and Skamarock*, 2002] that solves only for these fast modes on a small time step, while slower modes (e.g., advection) are solved on a larger time step.

**Figure 4:**
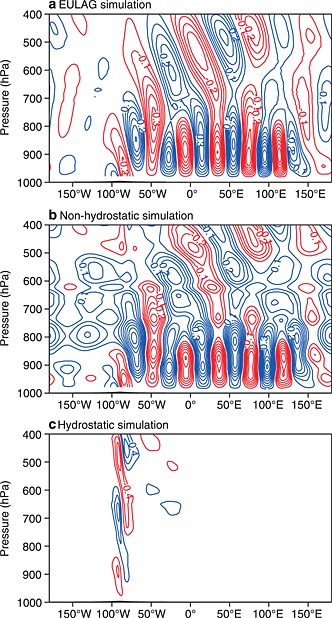
Vertical velocity in an idealized linearly sheared flow passed a quasi 2‐D hill with the nonhydrostatic (a) EULAG model and the corresponding (b) nonhydrostatic and (c) hydrostatic IFS simulations [*Wedi and Smolarkiewicz*, 2009]. IFS is the numerical model used at ECMWF, Reading, for midrange and seasonal forecasts. As demonstrated here, the hydrostatic version of IFS fails to simulate horizontally propagating gravity waves and the need for nonhydrostatic formulations becomes obvious. “©[2010 ECMWF]” Copyright belongs to the European Centre for Medium‐Range Weather Forecasting (ECMWF).

The importance of using a nonhydrostatic model to simulate gravity waves in a CPM was illustrated by, e.g., *Wedi and Smolarkiewicz* [2009]. They simulated an idealized flow over a mountain using an anelastic solver [*Prusa et al.*, 2008], and the nonhydrostatic and hydrostatic versions of European Centre for Medium‐Range Weather Forecasts (ECMWF)'s Integrated Forecast System model (see Figure 4). The nonhydrostatic version is able to simulate the horizontal propagation of gravity waves (nonhydrostatic lee waves) [see *Wurtele et al.*, 1987] as in the anelastic reference model, while the hydrostatic version only produces vertically propagating waves.

Additional modifications in the dynamics of CPMs might be necessary to improve model accuracy and stability. This is particularly important for CPM simulations in regions with complex orography. Even though the commonly applied terrain‐following vertical coordinates simplify the lower boundary condition, the applied mapping leads to spurious circulations over steep terrain due to discretization errors in the horizontal pressure gradient [*Mahrer*, 1984; *Klemp et al.*, 2003]. Improvements are obtained by defining a fixed and uniform hydrostatic background pressure and by evaluating the gradient only for the perturbation pressure [*Dudhia*, 1993]. Additional improvement was yielded by reformulating the terrain‐following coordinate [*Schär et al.*, 2002] such that model levels approach truly horizontal surfaces faster with height. Other modifications to, e.g., divergence damping and the lower boundary condition, have been described for the Consortium for Small‐Scale Modeling (COSMO) in Climate Mode (COSMO‐CLM) model by *Baldauf* [2013].

Finite difference discretization (compared to spectral methods) implicitly diffuse the prognostic variables at scales ranging from two horizontal grid spacings (Δ*x*) to about eight horizontal grid spacings (Δ*x*). This reduces the effective resolution of such models (see section 6.1 or *Ogaja and Will* [2014], *Denis et al.* [2002], and *Skamarock*, [2004]). This waste of computing resources is undesirable especially in computationally expensive CPM climate simulations. Using higher‐order numerical approximations could significantly increase the effective resolution [*Ogaja and Will*, 2014; *Ghosal*, 1996] and thereby reduce the computational costs of CPM simulations.

### 5.2 Parameterization of Clouds, Aerosols, and Radiation

Since convection parameterization schemes are not used in convection‐permitting models (CPMs), cloud microphysical processes and processes that contribute to the explicit triggering of deep convection on the grid gain in relevance. Microphysical processes in convective clouds are much more complicated than in stratiform clouds [e.g., *Pruppacher et al.*, 1998] because the stronger upward motion in convective clouds supports mixed‐phase processes and wider spectra of hydrometeor types. Cloud processes can be better represented by introducing additional hydrometeor species such as graupel or hail. They have faster fall speeds than, e.g., snow, and thus allow for less overall melting or evaporation [e.g., *Adams‐Selin et al.*, 2013].

Several studies examined the effect of including graupel or hail to the microphysics scheme of convection‐permitting models (CPMs). It is not clear if introducing graupel or hail weakens [*Van Den Heever and Cotton*, 2004; *Cohen and McCaul*, 2006; *Adams‐Selin et al.*, 2013] or strengthens [*Van Weverberg et al.*, 2011, 2012; *Morrison and Milbrandt*, 2010] cold pools formed from thunderstorm outflows. *Adams‐Selin et al.* [2013] found that simulations without graupel result in cold pools that are initially weaker because of the reduced cooling by the slowly falling snow, but they increase in strength over time. According to *Brisson et al.* [2015] (see Table 1, domain e), including graupel among the hydrometeors reduces the underestimation of summer precipitation. Interactions of newly introduced hydrometeors, such as graupel, with the radiative transfer should be introduced, but the different radiative properties of hydrometeors are commonly ignored [e.g., *Li et al.*, 2014].

*Van Weverberg et al.* [2014] compared one‐moment against two‐moment microphysics scheme (version of the *Seifert and Beheng* [2006]) in CPM simulations of 20 extreme case studies over Belgium. In a computationally more expensive two‐moment scheme, the number concentration and the mass of cloud water, ice, rain, snow, graupel, and hail are predicted, while in a one‐moment scheme the number concentration is prescribed. They found that precipitation is similarly simulated in the two approaches because of counteracting processes in the two‐moment scheme (size sorting of particles [*Chow et al.*, 2012] and collisional drop breakup [e.g., *Seifert et al.*, 2005]). Extreme precipitation in the simulations with a two‐moment scheme was found to be very sensitive to the treatment of drop breakup and the shape of the particle size distributions. Even though processes are being parameterized with more physical intuition, two‐moment schemes remain highly tunable and many depicted processes such as drop‐drop interactions and breakup [*Villermaux and Bossa*, 2009; *Giangrande et al.*, 2012; *Morrison et al.*, 2012; *Prat and Barros*, 2009], the formation of ice particles, and cloud interactions with aerosols are not well understood (mainly due to missing observations [e.g., *Rosenfeld et al.*, 2014]) and limit their potential.

The limited knowledge about these processes and their interactions can have far‐reaching consequences. In particular, the representation of cloud‐radiative feedbacks relies heavily on accurate representations of cloud cover and cloud‐radiative properties (e.g., ice versus liquid clouds). Most importantly, the parameterization of cloud‐aerosol interactions remains poorly understood and a key uncertainty in both global models and CPMs [e.g., *Tao et al.*, 2012]. As an example, the number of cloud droplets in polluted regions has been found to depend on the updraft speed in clouds [*Karydis et al.*, 2012]. As discussed in section 3, CPMs had been argued by some authors to not represent well the maximum speeds in clouds even though the bulk impact of an ensemble of clouds to the larger scales seems well represented. Thus, droplet number concentration and radiative properties of clouds [e.g., *Koren et al.*, 2010] might be misrepresented in CPMs with possibly far‐reaching consequences on the dynamics of clouds, e.g., cloud top height, extent, or aggregation, and the resulting precipitation [e.g., *Fan et al.*, 2007; *Ekman et al.*, 2004, 2006; *Lee et al.*, 2009].

Because of the widespread effect of aerosols on the climate system, it is important to improve the prescription of aerosols in climate models. Therefore, coupling CPM with aerosol modules that are able to model properties such as particle size, chemical composition, and mixing state [*Tao et al.*, 2012] would be beneficial. CPM in combination with two‐moment microphysics schemes have large potentials to improve the simulation of interactions between aerosols and clouds [*Rosenfeld et al.*, 2014; *Wang et al.*, 2011], but a better understanding of the underlying processes and their interaction with the resolved cumulus dynamics in CPMs is needed. For a more detailed description of the interaction of aerosols and clouds see *Levin and W. R. Cotton* [2008] and *Tao et al.* [2012, and references therein].

Not only convective precipitation is sensitive to the representation of cloud microphysics. *Liu et al.* [2011] (Table 1, domain a) investigated the sensitivity of wintertime precipitation in CPM climate simulations due to physical parameterizations. They showed that orographically enhanced precipitation is highly sensitive to the applied cloud microphysics parameterization with differences in average precipitation of up to 60%. The two applied two‐moment microphysics schemes were found to be superior to other schemes that clearly overestimated the snowfall amount.

In summary, sensitivities related to cloud microphysical parameterizations (and the related sensitivity of the cloud‐radiative feedback) remain considerably larger than those due to a mesh refinement at kilometer scales [*Wang et al.*, 2009; *Roh and Satoh*, 2014]. A more in depth discussion about modeling of clouds and microphysics can be found in *Tao and Moncrieff* [2009] or textbooks such as *Straka* [2009].

Since CPM climate simulations have a more realistic representation of orography, topographic shading, and differential heating in narrow valleys can be simulated. Nonhydrostatic climate models, such as the COSMO‐CLM and the Weather Research and Forecasting Model (WRF) offer an option to include orographic shading and slope effects on radiative transfer. Including those effects delays the breakup of valley inversion layers [*Colette et al.*, 2003], affects the buildup and melting of snowpack, and therefore alters the runoff [*Gu et al.*, 2012; *Liou et al.*, 2013]. However, the effect of topographic shading in real‐case simulations and on climatological time scales still remains largely unexplored.

### 5.3 The Parameterization of Turbulent Fluxes

The planetary boundary layer is the part of the atmosphere that is directly influenced by the Earth's surface and where turbulent processes act on time scales shorter than 1 h [e.g., *Stull*, 1988]. Turbulent fluxes of heat, water mass, and momentum are crucial within the boundary layer of the atmosphere. Even above the planetary boundary layer turbulence plays a critical role where ever the production of turbulent kinetic energy by buoyancy forces or by shear are significant. Outside of the planetary boundary layer, turbulent mixing can be of relevance near the tropopause where the jet stream induces wind shear or also in buoyant convective clouds which are generally highly turbulent. Most of the turbulent energy remains unresolved on grids applied in CPM simulations, and the unresolved fluxes thus have to be parameterized to incorporate their effect on the mean grid‐scale variables [*Bryan et al.*, 2003].

Basically, two conceptually different approaches for turbulence parameterizations might be considered relevant to CPM simulations. Mesoscale models and GCM solve for the ensemble‐averaged Navier‐Stokes equations, and the mean flow can be considered laminar. All turbulent kinetic energy and all turbulent fluxes remain unresolved and need to be parameterized. The grid spacing and the effective resolution (typically around seven horizontal grid spacing (Δ*x*)) [e.g., *Skamarock*, 2004] are large compared to the energy‐containing turbulent scales. In the other approach, traditional large‐eddy simulations [*Smagorinsky*, 1963; *Lilly*, 1967; *Deardorff*, 1974], the unsteady and anisotropic energy‐ and flux‐containing scales are resolved by choosing adequately fine grid spacings. The subgrid‐scale fluxes are closed, most simply, by an eddy‐viscosity model. The grid spacing required to run large‐eddy simulations is on the order of 100 m [*Bryan et al.*, 2003] and currently imposes too large costs to be feasible for climate predictions.

**Figure 5:**
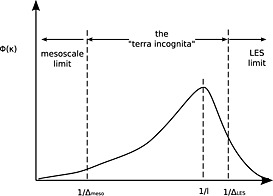
Simplified turbulence spectrum *ϕ*(*κ*) as a function of the horizontal wave number *κ*. The spectrum peaks at *κ* ∼ 1/*l* with *l* the length scale of the most energetic turbulent eddies (typically 1 km to 2 km over land [*Kaimal and Finnigan*, 1994]). Δ denotes the width of the spatial filter applied to the model equations. Mesoscale models typically operate at a filter width (Δ_meso_) that is large compared to *l*, while large‐eddy simulations (Δ_LES_) fully resolve the energy‐containing scale *l*. CPM climate simulations operate with grid spacings that fall into a range of scales termed as “terra incoginta.” The assumptions made in turbulence closures designed for the mesoscale limit and the large‐eddy simulation limit breakdown at these intermediate scales [*Wyngaard*, 2004, ©Copyright 2004 AMS].

CPM climate simulations operate at grid spacing that are neither coarse enough to fall into the mesoscale model regime nor fine enough to fall into the large‐eddy simulation regime (see Figure 5). The smallest horizontal grid spacings used so far are around 1 km [e.g., *Chan et al.*, 2014b, 2013; *Kendon et al.*, 2012, 2014]. This range of scales is close to the scale at which energy is produced by the largest turbulent motions, and both approaches discussed above are not designed for simulations at these scales [*Wyngaard*, 2004; *Bryan et al.*, 2003; *Zhou et al.*, 2014]. As outlined below, the design of turbulence schemes for this range of scales is ongoing and a relatively new research direction (at least in atmospheric sciences). For this reason, most CPM climate simulations currently use one of the mesoscale‐modeling approaches described below. This decision is mostly driven by historical and practical reasons. CPM climate simulations emerged from mesoscale modeling and thus inherited its parameterization approach. Although some studies report minor adaptions of their parameterization to the finer grid spacings [e.g., *Chan et al.*, 2013; *Kendon et al.*, 2012], the approach remains doubtful at these scales and the consequences and uncertainties of this decision for climate simulations are largely unknown.

A common way to parameterize the turbulent flux of a scalar in mesoscale models is to apply a local closure by relating the flux to the product of the vertical gradient of the resolved scalar and an eddy viscosity. Turbulence is assumed to be homogeneous in the horizontal and parametrizations are thus typically one dimensional [e.g., *Stull*, 1988; *Teixeira and Cheinet*, 2004]. Different approaches exist to close the problem. The eddy viscosities may be related to the local Richardson number [e.g., *Louis et al.*, 1982] or vertical profiles of these eddy viscosities may be obtained from similarity scaling [e.g., *Troen and Mahrt*, 1986]. Alternatively, a mixing‐length model can be used to relate these eddy viscosities to a characteristic mixing length [e.g., *Blackadar*, 1962] and a characteristic velocity scale. The latter may be obtained from a balance equation of the turbulent kinetic energy [*Kolmogorov*, 1942; *Prandtl*, 1945; *Mellor and Yamada*, 1982].

The observation that heat fluxes in the convective boundary layer are directed upward and thus against the slightly stable gradient in potential temperature lead to the development of nonlocal closures for turbulent fluxes. In contrast to local closures, the transilient nature of larger eddies and thermals is accounted for such that fluxes are no longer limited to small local eddies. Nonlocal transport results from both dry shallow thermals in the boundary layer [*Deardorff*, 1966; *Stull*, 1984] and from moist deep thermals in the free troposphere [*Romps and Kuang*, 2011]. For the dry convective boundary layer, local parameterizations have been corrected to account for these nonlocal effects [*Deardorff*, 1966; *Holtslag and Moeng*, 1991] or local closures have been replaced by mass flux closures [e.g., *Wang and Albrecht*, 1986; *Randall et al.*, 1992]—traditionally used for deep convection [*Arakawa*, 1969]. A combination of the two approaches has been proposed by *Siebesma et al.* [2007].

In the conventional approach using local closures, additional mass flux parameterizations have to be applied to account for the transport by shallow saturated thermals. The fact that it is an unsaturated thermal that later forms a saturated thermal aloft motivated an extension of *Siebesma et al.*'s [2007] combined approach to include the parameterization of shallow cumulus clouds [*Soares et al.*, 2004]. Such a unified approach has the advantage that an additional mass flux parameterization for shallow convection becomes obsolete. In general, unresolved shallow clouds need to be considered in CPMs also since they affect the cloud cover and total liquid water content which in turn affect radiative transfer. A representation of the subgrid‐scale cloud cover remains necessary even in CPMs, and a common approach is a statistical representation such as the one based on a Gaussian subgrid‐scale distributions of moisture [*Sommeria and Deardorff*, 1977].

Alternatively, given the joint distributions of subgrid‐scale fields allows for the reconstruction of turbulent fluxes. Once the joint distribution of, e.g., vertical velocity and moisture is known the unresolved moisture flux may be recovered by integrating over this joint distribution. In a similar way, the cloud cover can be determined. More recently, this technique was applied by *Golaz et al.* [2002] who assumed that joint distributions are characterized by the family of double Gaussian distributions. Their specific shape is determined by finding the best match to several statistical moments that are prognostic in the model. Based on a similar but simplified theoretical foundation, *Bogenschutz and Krueger* [2013] developed a subgrid‐scale parameterization for turbulence and shallow cumulus clouds for CPMs. A comparison with large‐eddy simulations shows that their parameterization is able to improve CPM simulations of, e.g., shallow cumulus clouds and their transition to deep convection. On top of that, the additional costs of their parameterization are reasonable.

As mentioned above, the energy spectrum of deep convective clouds is continuous across kilometer scales. The interaction of neighboring scales is thus significant and contributes to the turbulent fluxes. This issue has been realized in the engineering community [e.g., *Leonard*, 1974; *Bardina et al.*, 1980; *Chow et al.*, 2005], and it is the subject of ongoing research to design similar schemes for CPMs. As an example, *Moeng et al.*'s [2010] parameterization establishes a link between the largest unresolved eddies and the smallest resolved eddies. A priori tests of this scheme showed promising results for the parameterization of both horizontal and vertical fluxes in a deep convective scenario. Similarly, *Moeng* [2014] developed a mass flux model based on an updraft‐downdraft assumption and related the unresolved vertical fluxes to the horizontal gradients of resolved variables. However, both these promising developments have not yet been tested and used in CPM climate simulations.

Parameterizations of subgrid‐scale clouds are especially relevant also for stratiform clouds that form near sharp inversions at the top of the boundary layer. Issues in modeling such cloud sheets have been reported even from large‐eddy simulations since they underresolve the strength of the inversion at which these clouds form [*Stevens et al.*, 2003]. As long as these clouds remain unresolved, their parameterization remains key as the radiative effects of such warm cloud decks are considerable [*Hartmann et al.*, 1992; *Bony and Dufresne*, 2005].

To summarize, the parameterization of turbulent fluxes for CPMs is one of the key challenges for CPM simulations. New frameworks are currently being developed and tested to parameterize the unresolved transport related to dry and moist shallow convection and deep convection at kilometer scales. Currently, most CPMs still rely on one of the traditional parameterization approaches developed for boundary layer turbulence in mesoscale models.

### 5.4 Soil Processes and Soil‐Vegetation‐Atmosphere Coupling

In LSMs, large‐scale properties are used to parameterize deep convective transport. Since such parameterizations are not used in CPMs, other parameterizations that affect the small‐scale dynamics and thermodynamics at the grid‐scale gain importance (see section 5). Soil‐atmosphere interactions are important processes in this respect [e.g., *Pielke*, 2001]. Several studies showed that the soil moisture‐precipitation feedback dependents on the used convection parameterization in LSMs. CPM are able to simulate this feedback more realistically [e.g., *Hohenegger et al.*, 2009; *Froidevaux et al.*, 2014; *Taylor et al.*, 2013] (see section 6.2.4).

Furthermore, many processes in the soil or at the surface, which are highly nonlinear such as evaporative processes, are still not resolved in CPMs. Averaging parameters for calculating these processes in LSMs lead to biases [*Schomburg et al.*, 2010]. Thus, to account for the surface variability in atmospheric modeling, the tile approach, that subdivides the surface within an atmospheric grid column into several classes, might be an appropriate alternative to overcome this problem [*Ament and Simmer*, 2006]. Considering vegetation as a dynamic parameter might be another treatment to the land surface variability. By using a rather simple soil‐vegetation‐atmosphere model, *Tölle et al.* [2014] (see Table 1, domain f) showed that changing the vegetation parameters affects the shortwave radiation balance locally and the cloud cover regionally. They found that converting agricultural land to bioenergy plantations resulted in a local cooling due to increases of latent heat fluxes. A regional cooling occurs through increases in cloud cover. The local cooling effect was up to a factor of 25 greater than the regional cooling. Previous LSM studies reported of marginal changes in temperatures due to land cover transformations since coarser resolution models could not capture the local effect [e.g., *Brovkin et al.*, 2004; *Pitman et al.*, 2009; *Heck et al.*, 2001].

Even in state‐of‐the‐art CPMs, feedbacks between hydrologic subsurface models (e.g., simulating groundwater dynamics) with the land surface and atmosphere are generally neglected or oversimplified [e.g., *Zhou and Li*, 2011]. These simplifications may have a significant impact on the outcome of CPM climate simulations. Experimental and modeling studies demonstrated that in the critical zone, where the water table depth ranges between 10^0^ m and 10^1^ m from the land surface, even small changes in the depth of the water table may result in significant changes in soil moisture near the land surface and the associated latent and sensible heat fluxes [*Kollet and Maxwell*, 2008; *Szilagyi et al.*, 2013]. Such processes can maintain increased soil moisture values and latent heat fluxes under long periods without precipitation [*Alkhaier et al.*, 2012] or accelerate the depletion of moisture at the land surface due to declining water tables. While these are well‐studied processes, their role in two‐way feedbacks with the atmosphere has been investigate only relatively recently [e.g., *Maxwell et al.*, 2007]. In this context, the central hypothesis has been posed that these feedbacks may lead to changes in vertical wind velocities, boundary layer heights, and ultimately in essential climate variables, such as precipitation and air temperature at various space and time scales.

Based on the early blueprint for integrated hydrologic response models by *Freeze and Harlan* [1969], a number of integrated climate‐hydrologic model development efforts and simulation studies emerged. They connected the atmosphere with subsurface hydrologic models of various degrees of complexity but still in a more parameterized and computationally efficient way than the early proposal [*Gochis et al.*, 2013; *Maxwell et al.*, 2011; *Leung et al.*, 2011; *Miguez‐Macho et al.*, 2007; *Niu et al.*, 2007; *Shrestha et al.*, 2014; *York et al.*, 2002]. Numerically, more concise approaches require considerably more computational resources [e.g., *Maxwell et al.*, 2011; *Shrestha et al.*, 2014]. Since hydrologic processes of the soil are on scales of the order of 10^0^ m in the lateral and 10^−2^ m in the vertical direction [*Vogel and Ippisch*, 2008], there is still a large‐scale mismatch even if CPMs are used.

### 5.5 Initial Conditions

A challenging task for CPM climate simulations is the initialization of slow varying fields such as soil moisture and deep soil temperature. While the atmospheric component of climate models reaches an equilibrium state after a few days (spin‐up time) it can take several years for deep surface layer properties [e.g., *Cosgrove et al.*, 2003]. Due to the high computational costs of CPM climate simulations it is not feasible to run the model until the soil spun‐up. A common approach is to use interpolated climatological mean soil conditions from a long‐term LSM simulation to initialize the CPM or to start the CPM simulation a few months earlier to let the model spin‐up [e.g., *Prein et al.*, 2013a; *Rasmussen et al.*, 2014; *Ban et al.*, 2014]. The magnitude and consequences of errors introduced by either of the two approaches for CPM climate simulation are unknown.

Even longer spin‐up times are needed for coupled ocean models. In coupled GCM ocean models, deep ocean layers require hundreds of years to adjust while the upper ocean only requires about 50 years [*Kantha and Clayson*, 2000]. Methods such as data assimilation (observations are used to derive initial conditions) [e.g., *Carton and Giese*, 2008] could help to reduce the spin‐up time. None of the reviewed CPM climate simulations uses a coupled ocean model. Instead, sea surface temperatures are taken from the coarse‐scale driving models (reanalysis or GCM). The effects of the resulting scale difference between the highly resolved atmosphere and the coarsely resolved ocean or the consequences of the missing ocean‐atmosphere interactions on CPM climate simulations is unexplored.

### 5.6 The Importance of External Parameters

To explore the full potential of CPM climate simulations, high gridded surface fields are necessary. Land cover information is typically provided on grids between 1 km and 300 m [*Loveland et al.*, 2000; *Masson et al.*, 2003; *Arino et al.*, 2008] that are sufficiently small for CPM. However, soil properties, such as soil type or soil texture, are typically given on grids ≥10 km [e.g., *Sanchez et al.*, 2009] and contain large uncertainties in some regions [e.g., *Sanchez et al.*, 2009]. Also, new land use classes such as urban land types and smaller lakes can be important as they are resolved by CPMs. Some regional climate models (RCMs) include specific lake models to better account for the effect of lakes on the regional climate [e.g., *Mironov*, 2003; *Kumar et al.*, 2008; *Subin et al.*, 2012; *Martynov et al.*, 2012] or urban models (see section 6.7).

Another precondition for CPMs are highly resolved digital elevation models. State‐of‐the‐art digital elevation models have sufficiently high grid spacing between 1 km and 30 m [*Hastings and Dunbar*, 1998; *Suwandana et al.*, 2012]. Care has to be taken because digital elevation models can contain horizontal shifts and large isolated peaks in mountainous regions [*Messmer and Bettems*, 2013; *Suwandana et al.*, 2012]. It is advisable to compare the digital elevation model of CPMs to those used in highly resolved observational data sets or other CPM simulations.

### 5.7 Computational Aspects and Big Data Handling

CPM climate simulations pose a number of high‐performance scientific computing challenges. The continuous weather and climate model development that makes CPM climate simulations possible is concurrent to considerable progress of highly scalable supercomputing infrastructure with high‐bandwidth and low‐latency network connections (interconnects), multicore CPUs, or parallel file systems leading to ever‐increasing computational resources [*Navarra et al.*, 2010; *Smari et al.*, 2013]. The cumulative peak performance was about 250,000 times higher in 2014 than it was in 1993 (http://www.top500.org).

Primarily, due to their small horizontal grid spacing, which demands small time steps below 60 s in combination with many model levels (typically more than 50), and domain sizes that may range from overall 8×10^6^ grid elements, e.g., for mesoscale river catchments to up to more than 130×10^6^ for continental domains, CPM climate simulations are computationally very demanding and require specific approaches and solutions that also enable them to efficiently run on next‐generation exascale computing systems (>10^18^ floating point operations per second) [*Attig et al.*, 2011; *Keyes*, 2011; *Davis et al.*, 2012]. With large continental model domains, CPM climate simulations show already good scaling behavior and high parallel efficiency up to a few thousand parallel processes [*Michalakes et al.*, 2008].

**Figure 6:**
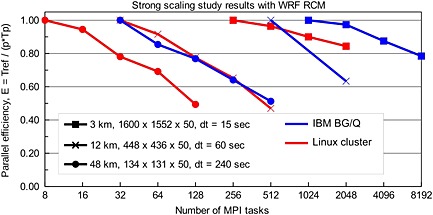
Example of real‐data strong scaling studies with WRF RCM on a massively parallel IBM BG/Q HPC system (blue) and a standard Linux cluster with Infiniband interconnects (red) for different pan‐European Coordinated Regional Downscaling Experiment (CORDEX) model domains at about 48 km resolution (EUR‐44, circle), 12 km (EUR‐11, cross), and a 3 km CPM domain (squares). For the individual scaling experiments, the parallel efficiency (*E*) is given in relation to the number of parallel tasks (or CPU cores). *E* is defined as the reference runtime with a specific number of parallel tasks (Tref) divided by the runtime with an increased number of tasks (Tp) and divided by the fractional increase in task number (*p*). A linear speedup (i.e., Tref/Tp = *p*) would lead to a sustained efficiency equal to 1. The grid elements in *x*, *y*, and *z* direction as well as the model's time step (d*t*) are given in the inscribed box. Increasing the number of CPUs by a factor of 8 reduces the computational efficiency by 50%, while for large domains the reduction is only about 20%.

The speedup (relation between the serial and parallel runtime) and parallel efficiency (speedup per additional processor) are measures to evaluate the success of a parallelization effort of an application, for example, in strong scaling studies with a constant problem size during the experiment. Figure 6 shows examples for the parallel efficiency of CPM model runs for different pan‐European model domains. Increasing the number of CPUs by a factor of 8 in simulation with a small computational grid (e.g., in the 48 km simulation, circles) leads to a reduced computational efficiency by about 50% due to the communication overhead in the parallel simulation. For large‐domain sizes (e.g., 3 km simulation, squares) the reduction is only about 20%, whereas it can be seen that the RCM can nicely scale to 8192 tasks if the domain size is sufficiently large. There are specific profiling tools that can help to find performance bottlenecks to speed up the runtime of CPM climate simulations [e.g., *Geimer et al.*, 2010; *Carns et al.*, 2011].

Today's high‐performance computing systems are massively parallel distributed memory multicore supercomputers with very fast communication networks for data exchange between the individual compute nodes with a shared memory [e.g., *Geer*, 2005]. Strategies to improve the efficiency of CPM climate simulations on modern high‐performance computing systems include hybrid parallelization combining Message Passing Interface (MPI) and OpenMP communication protocols [e.g., *Michalakes et al.*, 2004; *Jin et al.*, 2011] or simultaneous multithreading with up to four independent threads running on a single core [e.g., *Michalakes et al.*, 2004; *Jin et al.*, 2011].

A development in supercomputing that seems especially relevant for highly scalable resource‐intensive CPM climate simulations is the evolution of hybrid or heterogeneous high‐performance computing architectures where multicore CPUs are combined with accelerators (either graphic processing units (GPUs) or Many Integrated Core chip designs) on a single compute node [*Brodtkorb et al.*, 2010; *Liu et al.*, 2012]. Especially the energy efficiency of the accelerated systems makes this architecture suitable for future exascale systems [*Davis et al.*, 2012]. However, irrespective of the choice and availability of hardware, standard MPI‐parallelized codes commonly used in CPMs may need substantial porting, profiling, tuning, and refactoring, i.e., restructuring, to efficiently perform on these new architectures [*Hwu*, 2014]. Optimized WRF code with few code changes is reported to show a speedup of 1.4X at Δ*x* = 2.5 km for a continental U.S. model domain [*Meadows*, 2012]. *Michalakes and Vachharajani* [2008] reach an overall speedup of 1.3X after adapting specific parts of the WRF model. The COSMO model has been transferred into a GPU implementation where physical parameterizations show typical speedups between 3X and 7X [*Lapillonne and Fuhrer*, 2014].

The small spatial grid spacing of the CPM climate simulations and necessary frequent output intervals (typically 1 h to resolve, for example, the diurnal cycle adequately) combined with increasing ensemble sizes pose a substantial big data challenge [*Aloisio and Fiore*, 2009]. For example, storing 5 daily plus 43 subdaily surface variables plus four 3‐D variables at 50 model levels at hourly output intervals yields for continental‐scale model domains (1600 times 1552 grid cells) a data volume of about 1.7 Tb month^−1^, i.e., 204 Tb for a single decadal run. Data input and output operations, handling and transfer, and analysis as well as storage and archival of such data volumes therefore become a grand challenge [*Overpeck et al.*, 2011], especially as storage and network (i.e., input and output bandwidth) developments are lacking behind the compute performance developments. Possible solution to this problem are to use parallel input and output, to fine‐tune input and output settings, to transfer model outputs to data types with a reduced numerical precision, to apply compression and feature reduction techniques [*NAFEMS World Congress*, 2009; *Clyne and Norton*, 2013], or to perform data analysis and visualization in situ with the simulation [*Zhang et al.*, 2012; *Childs et al.*, 2013].

Generally, efficient generic analysis frameworks for big geoscience data are still rare, leading to a disparity between parallel, highly scalable model simulations and file systems versus serial analysis/postprocessing tools and often still serial input and output [*Steed et al.*, 2013].

### 5.8 Model Evaluation

Many of the above described model developments, the evaluation, and assignment of added value in CPM climate simulations are crucially dependent on high‐quality subdaily observational data sets with grid spacings at the kilometer‐scale. The high temporal and spatial resolution is needed to examine the simulation of small‐scale extreme events; an area where CPM climate simulations have high potentials to improve LSM simulations (see section 6). Globally, there are only a few regions such as the European Alps (Wegener Net [*Kirchengast et al.*, 2014], INCA [*Haiden et al.*, 2011], the German Bight and Northern Germany (F. Feser and B. Schaaf, personal communication, 2014), Switzerland (RdisaggH [*Wüest et al.*, 2010]), the United Kingdom (NIMROD [*Golding*, 1998]), or the U.S. [*Lin and Mitchell*, 2005]) where such kind of data is available for at least several years. Especially the combination of radar and surface data can provide observations in high spatial and temporal resolutions. Such data sets are already provided in some regions (e.g., European Alps, UK, and U.S.). Also, new approaches to derive atmospheric observations such as the usage of cellular communication networks [*Overeem et al.*, 2013] or sensors in mobile phones [*Mass and Madaus*, 2014] could be valuable for evaluating CPM climate simulations in future. Satellite data sets [e.g., *Smith et al.*, 2007] are very promising because they have global coverage and are independent from surface‐based measurements. However, often the limiting factors of these rather new approaches are the length of the observational record, the low accuracy, the high measurement errors, the drifts in the data set, and the data inhomogeneities.

Alternatives for model development are observations derived from measurement campaigns like the Mesoscale Alpine Programme [*Bougeault et al.*, 2001], the Terrain‐Induced Rotor Experiment [*Grubišic et al.*, 2008], or the Initiation of Convection and the Microphysical Properties of Clouds in Orographic Terrain (Convective And Orographically‐Induced Precipitation Study [*Wulfmeyer et al.*, 2011]). However, they typically last only for a few weeks which is insufficient for evaluating climate simulations.

## 6 The Added Value of CPM Climate Simulations

### 6.1 Potential Added Value

As already discussed in section 3, climate simulations have the advantage of avoiding error‐prone convection parameterizations by resolving deep convection explicitly and of improving the representation of orography and other surface forcing. These two basic features already give us hints of where added value of CPM climate simulations potentially exists: where/when deep convection is a dominant process (e.g., tropics, subtropics, and midlatitude summer) and in regions with strong spatial heterogeneities (e.g., mountainous regions, coastlines, and urban areas).

**Figure 7:**
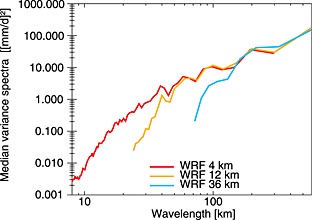
Median variance spectra for surface precipitation obtained from heavy precipitation events simulated in three 8 yearlong WRF simulations (red line Δ*x* = 4 km, yellow line Δ*x* = 12 km, and blue line Δ*x* = 36 km) in the headwater region of the Colorado River during December, January, and February (DJF). Both axes are logarithmically scaled (adapted from *Prein et al.* [2013b]). The spectra shows that the 4 km simulation has higher variances (potential added value) in short wavelengths, while the spectra are similar for wavelengths above approximately 100 km. ©Copyright 2013 AMS.

A useful tool to investigate the potential added value is the decomposition of the variance of atmospheric fields into contributions from different wavelengths [e.g., *Denis et al.*, 2002]. A representative example of a variance spectrum for wintertime heavy precipitation events simulated by a CPM climate simulations (Δ*x* = 4 km) and with two LSMs is shown in Figure 7. The largest differences between the CPM climate simulation and the LSM simulations are found for small wavelengths where the CPM has clearly higher variability. For larger wavelengths, however, the spectra of the different simulations start to converge. The wavelength on which the spectra of, e.g., the 12 km simulation starts to differ from the spectra of the CPM, is denoted as the effective resolution of the simulation (in this example 50 km, which is ∼4Δ*x*). The higher variability at small wavelengths is a precondition for potential added value in CPM climate simulations, but no guaranty. The form of the spectra also indicates that spatial or temporal averaging of the CPM climate simulation's precipitation will smooth out the potential added value. The variance spectra of other atmospheric variables have similar characteristics [e.g., *Skamarock*, 2004; *Horvath et al.*, 2011]. Alternative methods to analyze the spatial dependency in model fields are variograms or correlograms like applied in *Prein et al.* [2013b].

### 6.2 Precipitation

This section gives an overview of studies that have evaluated precipitation in CPM climate simulations against LSMs and observations. The observations used for validation are fine‐gridded precipitation data sets, mostly based on radar and rain gauge measurements. Thus, during the evaluation one should account also for the uncertainties in the observations [*Frei and Schär*, 1998; *Isotta et al.*, 2014].

#### 6.2.1 Diurnal Cycle

**Figure 8:**
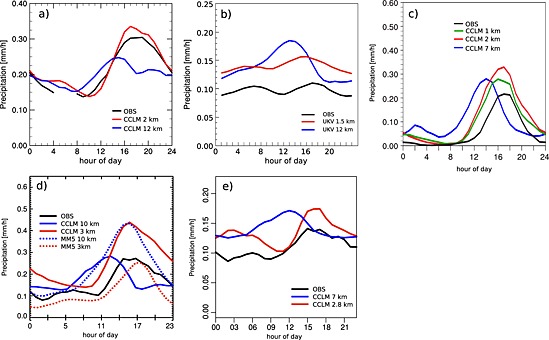
Mean diurnal cycle of (a) precipitation averaged across June, July, and August (JJA) in Switzerland (Table 1, domain m) [*Ban et al.*, 2014, ©2014. American Geophysical Union. All Rights Reserved]; (b) annually in Southern UK (Table 1, domain d) [*Kendon et al.*, 2012, ©Copyright 2012 AMS]; (c) July 2006 in Switzerland (Table 1, domain m) [*Langhans et al.*, 2013, ©Copyright 2013 AMS]; (d) June, July, and August (JJA) in eastern part of the Alps (Table 1, domain l) [*Prein et al.*, 2013a]; and (e) June, July, and August (JJA) in Baden‐Württemberg, Germany (Table 1, domain j) [*Fosser et al.*, 2014]. All CPM climate simulations show improvements in the shape (onset and peak) of the precipitation diurnal cycle compared to their corresponding LSM simulations.

A major added value using CPMs is the improved diurnal cycle of summer precipitation. Figure 8 shows the simulated diurnal cycles of precipitation over Switzerland (a and c), the Southern UK (b), the Eastern European Alps (d), and Baden‐Württemberg in Germany (e). The CPMs clearly improve the simulation of the onset and peak of the precipitation diurnal cycle in all investigated regions and models. Decreasing the grid spacing from 2.2 km to 1.1 km slightly reduces the overestimation of peak precipitation in the afternoon (Figure 8c) [*Langhans et al.*, 2013]. The LSMs have a poor representation of the diurnal cycle of precipitation due to the use of convection parameterizations rather than unresolved topography [*Langhans et al.*, 2013; *Prein et al.*, 2013a].

*Fosser et al.* [2014] (see Table 1, domain j) investigated the physical processes leading to improvements in the diurnal cycle of convective precipitation. They showed that the maximum atmospheric instability occurs before convective precipitation only when convection is not parameterized, which is in good agreement with results from numerical weather forecast [e.g., *Baldauf et al.*, 2011]. Moreover, small differences in the vertical profiles of temperature and humidity lead to systematically higher levels of cloud formation and free convection in CPM compared to LSM, which can lead to deep convection and explain the higher hourly precipitation intensities in CPM [*Fosser et al.*, 2014].

Orographic lifting can play an important role in triggering convection over complex terrain [e.g., *Kalthoff et al.*, 2009; *Khodayar et al.*, 2013; *Houze*, 2012]. In the European Alps, the daily anticorrelation between upvalley flow and rain rate is well represented in the CPM simulation of *Langhans et al.* [2013], while this dynamical response to convective precipitation is missing in the LSM simulation as upvalley winds endure during the convective event [*Langhans et al.*, 2013]. In the Black Forest in Germany, higher spatial resolution leads to areas of convergence and divergence that are more consistent with the underlying orography as well as to higher values of vertical velocity [*Barthlott et al.*, 2006; *Fosser et al.*, 2014].

#### 6.2.2 Spatial Patterns

Analyzing the spatial patterns of hindcast CPM climate simulations in high spatial and temporal resolutions is challenging because of small spatial and temporal shifts between the observed and modeled precipitation. This so‐called “double penalty” problem [e.g., *Wernli et al.*, 2008; *Prein et al.*, 2013a] can be avoided by using special statistical methods that account for such displacements (a summary of such methods can be found in *Prein and Gobiet* [2011]). Applying two of these methods, *Prein et al.* [2013a] (Table 1, domain l) showed improvements in the distributions of hourly precipitation patterns and more realistic (smaller and more peaked) precipitation objects. This is in line with results from numerical weather prediction forecast [e.g., *Wernli et al.*, 2008; *Roberts and Lean*, 2008]. The smaller and denser precipitation objects as well as a more realistic simulation of the diurnal cycle of precipitation tend to increase global radiation at the surface in CPM climate simulations (see section 6.4).

*Prein et al.* [2013b] (see Table 1, domain a) used correlograms and variograms to investigate spatial patterns of mean and extreme precipitation in the Colorado Rocky Mountains. They found improvements in spatial correlation and variability structures for spatial scales smaller than 100 km. Above this scale spatial patterns are similar to those of their LSMs.

#### 6.2.3 Biases in Mean and Extreme Precipitation

There is no general improvement in daily mean precipitation between CPMs and LSMs [e.g., *Prein et al.*, 2013a; *Ban et al.*, 2014; *Fosser et al.*, 2014; *Chan et al.*, 2013]. Improvements in simulating daily precipitation intensity seem to be dependent on the region and models since *Ban et al.* [2014] and *Prein et al.* [2013b] found improvements while *Fosser et al.* [2014] and *Chan et al.* [2013] did not.

CPMs have a tendency for too intense daily heavy precipitation in mountainous regions [*Kendon et al.*, 2012; *Langhans et al.*, 2013; *Prein et al.*, 2013a; *Ban et al.*, 2014] that might be related to observational uncertainties [*Frei and Schär*, 1998; *Isotta et al.*, 2014] or the still too coarse horizontal grid spacing (Δ*x*) of the applied CPM. However, *Langhans et al.* [2012a] found no significant differences in precipitation magnitudes in a 550 m run in comparison to a 2.2 km run in Switzerland. In addition, the microphysics might not be well tuned for CPMs (see section 5.2). More consistent improvements have been found for the simulation of wet‐day frequencies [*Ban et al.*, 2014; *Fosser et al.*, 2014].

**Figure 9:**
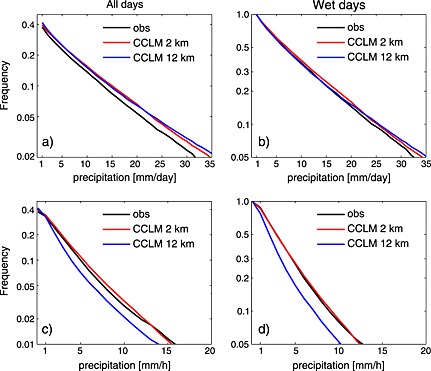
Cumulative distributions of (a, b) daily precipitation and (c, d) daily maximum 1 h precipitation as a function of threshold, expressed relative to the total number of days shown in Figures 9a and 9c and relative to the number of wet days shown in Figures 9b and 9d for the data at 24 Swiss stations. The distributions have been calculated for June, July, and August (JJA) in the period 1998–2007 [*Ban et al.*, 2014]. The CPM simulation with Δ*x* = 2 km reproduces the observations very well, while the LSM simulation with Δ*x* = 12 km underestimates the frequency of daily maximum 1 h precipitation. ©2014. American Geophysical Union. All Rights Reserved.

The largest differences between LSM and CPM climate simulations occur on short (subdaily) time scale and for summertime high‐precipitation intensities. Heavy hourly precipitation is typically underestimated in LSM, while large improvements were found in CPM climate simulations [*Ban et al.*, 2014; *Fosser et al.*, 2014; *Chan et al.*, 2014b, 2013] (see Figure 9, for an example). *Gensini and Mote* [2014] (see Table 1, domain c) show that CPM climate simulations can reproduce proxies for hazardous convective weather (tornadoes, thunderstorm, and large hail) in the USA.

According to the Clausius‐Clapeyron relation, the saturation vapor pressure in the atmosphere increases at a rate of 7% per degree, and thus, it is expected that extreme precipitation will increase at the same rate [*Allen and Ingram*, 2002; *Trenberth et al.*, 2003]. Indeed, the observations show that extreme hourly precipitation increases at a rate of 7% per degree for temperatures below 12°C, but above that threshold it exceeds the expectations from the Clausius‐Clapeyron relation [*Lenderink and Van Meijgaard*, 2008, 2010; *Attema et al.*, 2014; *Mishra et al.*, 2014; *Hardwick Jones et al.*, 2010; *Westra et al.*, 2014]. *Ban et al.* [2014] have shown that their CPM simulations reproduced the observed scaling across Switzerland, while their LSM had difficulties in reproducing the observed scaling, especially in a region with complex topography.

During wintertime, precipitation pattern mainly gets improved due to the better resolved orography in CPMs. *Ikeda et al.* [2010] and *Rasmussen et al.* [2011] (see Table 1, domain a) showed improvements of simulated snowpack at grid spacing smaller than 6 km due to the improved representation of mesoscale orographic forcing in the headwater region of the Colorado River. LSMs tend to spread precipitation horizontally as a result of broader and weaker updrafts.

#### 6.2.4 Soil Moisture‐Precipitation Feedback

Climate‐relevant feedback mechanisms revealed not only different magnitudes but even different signs depending on whether a LSM or a CPM was used. For example, CPM climate simulations in *Hohenegger et al.* [2009] show a negative soil moisture‐precipitation feedback; i.e., wetter soil leads to less precipitation onto these areas. This is in conflict with results from LSMs which show mostly a positive soil moisture‐precipitation correlation.

*Froidevaux et al.* [2014] demonstrate positive and negative feedbacks in case of varying ambient wind conditions utilizing numerical experiments. For example, convective precipitation may be initiated over dry cells and enhanced while propagating over wet soil patches leading to a positive feedback. Similar findings are reported in a study of *Taylor et al.* [2013], who investigated the soil moisture‐precipitation feedback in the Sahel.

### 6.3 Two Meter Temperature

CPM climate simulations are able to improve climatological mean temperature at a height of 2 m especially in mountainous regions during summer [*Hohenegger et al.*, 2008; *Prein et al.*, 2013a]. This added value can be mainly related to the better resolved orography that causes an increase in spatial variability of temperature at a height of 2 m. *Prein et al.* [2013a] (see Table 1, domain l) showed that applying a simple height correction (6.5 K/km) to their LSM simulations yields similar results. However, such kind of corrections cannot account for processes related to temperature at a height of 2 m such as snow accumulation or increased heating of the atmosphere. In winter this added value is smaller or not present at all probably because of a poor representation of inversions in the observational data set and the weak vertical mixing during this season.

Simulating inversions in CPM climate simulations usually demands subkilometer‐scale horizontal grid spacing (Δ*x*) and a high vertical resolution in the boundary layer because of the small‐scale processes involved. Short‐term convection‐permitting model (CPM) simulations are able to realistically simulate valley inversions and the related temperature and wind structures [*Zhong and Whiteman*, 2008; *Vosper et al.*, 2013; *Wei et al.*, 2013]. How CPM performs on climate time scales is unknown. This topic seems to be especially interesting because valley inversions are often decoupled from the free atmosphere, which means that these local climates may respond differently from the regional‐scale climate projected by LSMs [e.g., *Daly et al.*, 2010].

### 6.4 Cloud Cover and Energy Balance

In this section, the effects of CPM climate simulations on the cloud cover and surface energy balance is investigated. In summer, CPM climate simulations tend to increase incoming shortwave radiation by up to 30%, which is related to the decrease of cloud cover, a shift in the diurnal cycle of convection, and smaller and denser convective clouds in CPM [*Prein et al.*, 2013a; *Langhans et al.*, 2013; *Fosser et al.*, 2014]. During winter, the increase of shortwave radiation is less pronounced because of more stratiform clouds and different microphysical processes [*Ban et al.*, 2014; *Prein et al.*, 2013a]. How this additional energy is converted into sensible or latent heat fluxes at the surface is strongly dependent on the applied model [*Prein et al.*, 2013a].

Over central Europe, an overestimation of high cloud cover for LSM compared to brightness temperature observations was shown by *Böhme et al.* [2011] during 2 years of the operational numerical weather prediction runs. In contrast to climate simulations, the operational data assimilation cycle should decrease cloud cover biases. This overestimation was shown, also with COSMO‐CLM, for the summer season by *Langhans et al.* [2013] and M. Keller et al. (submitted manuscript, 2015). The bias is consistently decreased in CPM but is still positive [*Böhme et al.*, 2011; *Langhans et al.*, 2013; M. Keller et al., submitted manuscript, 2015]. Contrary to high cloud cover, low‐level and midlevel cloud covers have negative biases [*Langhans et al.*, 2013]. The high cloud cover bias can be reduced in the COSMO‐CLM CPM by applying a two‐moment microphysics scheme [*Seifert and Beheng*, 2006] with ice sedimentation instead of the standard one‐moment microphysics scheme without ice sedimentation M. Keller et al. (submitted manuscript, 2015). Related to the improvements in high cloud cover is also a reduction of the negative bias for top of the atmosphere outgoing longwave radiation in summertime CPM climate simulations.

### 6.5 Impact Modeling

CPM climate simulations are able to provide spatial data on scales that are small enough to derive impact‐relevant information.

In a pioneering study *Mölg and Kaser* [2011] (see Table 1, domain p) showed that it is possible to use CPM climate simulations output directly to simulate the energy and mass balance of glaciers. This enables them to avoid statistical adjustments of model output necessary with large‐scale model (LSM) climate simulations because of the scale mismatch with mountain glaciers where the typical processes are on the order of a few kilometers [*Machguth et al.*, 2009; *Kotlarski et al.*, 2010]. The approach of *Mölg and Kaser* [2011] allows to quantify the dynamical interaction between the atmosphere and cryosphere that operate on very different spatial and time scales in a full physical way. This direct coupling enables to study the influence of the dynamic, thermodynamic, and microphysics phenomena that are influenced by mountain‐induced flow on the mass balance of glaciers. This has the potential to enhance the understanding of processes related to glacier responses to climate forcing.

CPMs have been coupled with hydrology models to improve the forecast of river runoff and flooding [e.g., *Cloke and Pappenberger*, 2009; *Bartholmes and Todini*, 2005]. Several studies highlight the potential benefits of CPMs applied to flash flood predictions [*Cloke and Pappenberger*, 2009; *Renner et al.*, 2009]; however, very little is known about the real potentials of CPMs on the climate time scale.

A similar picture can be seen for other impact‐related research fields such as the simulation of renewable energy production (e.g., wind [e.g., *Foley et al.*, 2012], solar energy [e.g., *Kleissl*, 2013], or bioenergy plantations [e.g., *Tölle et al.*, 2014]) or the modeling of weather‐ and climate‐related economic risks [e.g., *Weitzman*, 2009] that potentially could highly benefit from CPM climate simulations.

### 6.6 Tropical Cyclones

Although tropical cyclones are synoptic‐scale objects with spatial extents of several hundreds to thousands of kilometers, small‐scale features such as deep convection or narrow wind systems are crucial for their formation and characteristics such as maximum wind speed and central pressure. Several studies indicate improvements in the simulation of tropical cyclones when CPMs are used [e.g., *Braun*, 2002; *Davis and Bosart*, 2001]. *Gentry and Lackmann* [2010] tested the sensitivity of simulating Hurricane Ivan (2004) on horizontal grid spacing (Δ*x*) with the WRF model. They found that decreasing horizontal grid spacing (Δ*x*) from 8 km to 1 km leads to a drop in minimum central pressure of 30 hPa attributed to small‐scale physical processes, which are important for tropical cyclone intensity. The domain size of the CPM should not be smaller than 500 km to simulate realistic small‐ and large‐scale features of the storm.

*Taraphdar et al.* [2014] compared simulations of Indian Ocean cyclones from a CPM at Δ*x* = 1.1 km with LSM simulations and found improvements in the observed tracks and intensities in their CPM simulations.

### 6.7 Urban Climate

The decrease in grid spacing of RCM to the CPM scale has important benefits for the representation of the local city climate. This opens the way for an improved assessment of the climate change impact due to urbanization. It is important that this impact is included in climate projections for the future: For example, for North America, urban‐induced climate change is of the same order of magnitude as that due to long‐lived greenhouse gases [*Seager et al.*, 2007].

For numerical weather prediction, the typical convection‐permitting model (CPM) spatial scale has already been reached a decade ago, implying that some grid cells are completely urbanized [*Gimeno et al.*, 2008]. Since then, the international community has been working on the implementation of urban schemes in atmospheric models [e.g., *Grossman‐Clarke et al.*, 2005; *Masson*, 2006]. This has led to an improved representation of the urban heat island in these models. The factors causing the urban heat island are nowadays well known with major contributions from the storage of heat during daytime and the release of heat during nighttime, the reduced evapotranspiration in cities, and the heat directly released by human activities.

One of the first to assess how urbanization affects climate in Europe was *Trusilova et al.* [2008] (Δ*x* = 10 km), indicating a reduced diurnal temperature range in urbanized regions. *Zhang et al.* [2010] confirmed this reduced diurnal temperature range for the Yangtze River Delta (China) and found precipitation increases of about 15% over urban or leeward areas in summer. Recent studies use grid spacing well below 4 km for specific metropolitan areas like Brussels [*Van Weverberg et al.*, 2008; *Hamdi and Vyver*, 2011], Phoenix [*Grossman‐Clarke et al.*, 2010], London [*Grawe et al.*, 2013; *Bohnenstengel et al.*, 2011], and Paris [*Wouters et al.*, 2013]. A comparison with observations has shown that the magnitude of the urban heat island is better represented at this resolution compared to the coarse scale (Δ*x* > 10 km) [*Trusilova et al.*, 2013]. In addition, the magnitude of the urban heat island is found to be larger at the CPM scale [*Wouters et al.*, 2013].

Urban climate models are used to investigate various heat‐stress mitigation and adaptation strategies including green urban infrastructure, high‐reflective surfaces, and thermal properties of building materials and vegetation [*Schubert and Grossman‐Clarke*, 2013]. *Taha* [2008] demonstrated a potential urban heat island mitigation for Sacramento of up to 3°C in response to increased albedo and urban vegetation cover. *Masson et al.* [2013] also show this potential of using lighter colors for building materials. They show that the albedo lowering, together with extending the nearby forests by 30%, substantially reduces the urban heat island thereby diminishing mortality during heat waves as well as the need for air conditioning for Paris. Models are currently further upgraded to take urban infrastructure into account, for example, by including enhanced water storage in tanks and water infiltration [*Coutts et al.*, 2013].

This brief overview shows a strong added value of applying CPMs for the simulation of the urban regions. The number of future urbanization scenarios is, however, quite limited and needs to be expanded in order to take into account the uncertainties associated with land use change in a similar way than the greenhouse gas scenarios. Increasingly, urban climate models are used as a tool for the assessment and efficiency of urban adaptation measures. It is expected that this trend will continue with more studies emerging that aim at the improving climate resilience of cities.

### 6.8 Improvements in Process Understanding

Since CPMs are able to model small‐scale processes such as deep convection, complex microphysics, or interactions of atmospheric flow with orographic, they are useful tools to study atmospheric processes and feedbacks that are difficult to observe. Examples are the investigation of soil‐atmosphere interactions (see section 6.2.4), the initialization and phenomenology of deep convection [e.g., *Khairoutdinov and Randall*, 2003; *Zhu et al.*, 2012; *Fosser et al.*, 2014], the study of local wind systems [e.g., *Schmidli and Rotunno*, 2010; *Schmidli et al.*, 2011], the scaling of precipitation with temperature (see section 6.2.3), the testing of various urban heat‐stress mitigation and adaptation strategies (see section 6.7), investigations of various climate change mitigation and adaption strategies through renewable energies [e.g., *Tölle et al.*, 2014], or changes in cloud microphysics and phase of surface precipitation due to climate change [*Mahoney et al.*, 2012].

Important to notice is, however, that the above mentioned process studies are mostly focusing on short time scales (from case studies to single years). Therefore, our understanding of how those processes are acting on climate time scales is very limited.

## 7 Influence of CPM Climate Simulations on the Climate Change Signal

As shown in the previous sections, CPM climate simulations facilitate the understanding of the climate system's behavior at scales most relevant to policy makers and citizens. Additionally, climate models are used to make projections of possible future evolutions of the climate system, typically up to 2100 [*IPCC*, 2013]. In this context we will investigate how a forcing on the global level (e.g., anthropogenic increases of greenhouse gases) will translate into changes on scales relevant to CPM climate simulations and where there are differences between climate projections with CPMs and LSMs.

Climate projections are based on possible future evolutions of the emissions of greenhouse gases, aerosols, and precursor gases as well as land use/land cover changes, which are derived based on socioeconomic and technological assumptions. As future anthropogenic choices cannot be predicted, different projections covering a range of possible scenarios are used to make projections for the future climate [*Nakicenovic and Swart*, 2000; *Van Vuuren et al.*, 2011; *Rogelj et al.*, 2012].

These scenarios are used to prescribe atmospheric composition in general circulation models, which in turn, simulate the response of the climate system to these changes. Due to their coarse grid spacings (typically between 100 km and 50 km [*IPCC*, 2013]) and the adapted physical parameterizations, these models are only capable of representing changes for large‐scale features like large‐scale frontal and storm systems. CPM climate simulations are expected to improve the representation of local features, such as wind systems dominated by topography [*Cholette et al.*, 2015] or feedback mechanisms like the soil moisture‐precipitation feedback [e.g., *Hohenegger et al.*, 2009].

### 7.1 Temperature Change Signal

*Junk et al.* [2014] and *Tölle et al.* [2014] show that the large‐scale temperature increase from GCMs can be downscaled by CPM climate simulations and that the better resolved orography increases the spatial variability of changes in temperature. *Tölle et al.* [2014] shows the potential for reducing the local climate change signal of maximum temperature by up to 20% if agricultural land is transformed by 10% to bioenergy plantations.

*Knote et al.* [2010] (see Table 1, domain g) conducted a CPM climate simulation for Western Germany and parts of Luxemburg. They found that the change signal of summertime temperature is dependent on the height of orography; i.e., the strongest warming of the daily minimum temperature is projected in the mountainous parts of their domain, whereas the strongest warming signal of daily maximum temperature occurs in the flat areas of the Rhine Valley.

**Figure 10:**
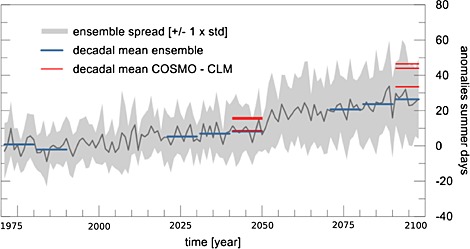
Anomalies of summer days (daily maximum >25°C) based on a multimodel ensemble (3 × 3 grid cells with Δ*x*=25 km each) data set for the period from 1971 to 2098 with respect to 1991–2000. The gray line shows the ensemble mean anomaly and the gray shaded area the ensemble spread. Blue lines indicate the decadal mean values of the anomalies of the ensemble data set. The two sets of three red lines represent decadal means for three selected artificial sites (average of (3 × 3 grid cells) of the Δ*x*=1.3 km data set) (adapted from *Junk et al.* [2014]). The CPM projects summer day temperature increases that are consistently above the LSM ensemble mean.

*Junk et al.* [2014] (see Table 1, domain i) compared climate indices indicating heat stress from a CPM climate simulations to the results of multi‐large‐scale model (LSM) ensemble for different sites in Luxembourg. Figure 10 shows that the CPM climate simulation anomalies for summer days (daily maximum >25°C) are higher than the multi‐large‐scale model (LSM) mean, but within the ensemble spread.

### 7.2 Changes in Precipitation and the Hydrological Cycle

**Figure 11:**
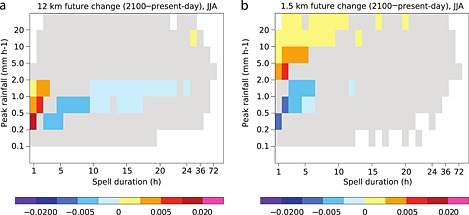
Simulated climatological difference in the joint distribution of wet spell duration and peak precipitation intensity for the southern UK and for June, July, and August (JJA) from (a) a 12 km model and (b) a 1.5 km model. The difference is computed between periods 1996–2009 and 2087–2099. Gray shaded areas show no significant differences at the 1% level. The CPM predicts an significant intensification of short‐duration extreme precipitation which is not projected by the LSM simulation. Reprinted by permission from Macmillan Publishers Ltd.: Nature Climate Change [*Kendon et al.*, 2014], copyright 2014.

Precipitation is another key variable that is expected to have major impacts on human societies, in particular due to its links to flooding. *Westra et al.* [2014] concluded that changes in frequency of urban and rural flash floods are driven by changes on subdaily rainfall. Present‐day global and regional climate models have limited ability to simulate subdaily precipitation extremes correctly as they do not explicitly resolve convective processes. *Kendon et al.* [2014] (see Table 1, domain d) compared the periods 1996–2009 with 2087–2099 using a high emission scenario and showed that in winter the precipitation climate change signals agree between the CPM and their LSM simulations. However, in summer the CPM simulations show intensification and higher frequencies of short‐duration (subdaily) heavy precipitation events that are not simulated by the LSM (see Figure 11). In a similar setup *Ban et al.* [2015] (see Table 1, domain m) performed convection‐permitting model (CPM) climate simulations over the European Alps for the period 1991–2000 and 2081–2090 using the same emission scenario as *Kendon et al.* [2014]. They found a weaker intensification of subdaily precipitation extremes than *Kendon et al.* [2014], which they attribute to the differences in the used models, investigated regions, and most important to the different analysis approach. A weaker intensification of very extreme precipitation intensities (precipitation intensities with longer return periods) has also been found by *Chan et al.* [2014a].

*Mahoney et al.* [2013] (see Table 1, domain b) selected an ensemble of summertime extreme precipitation cases from historical and future simulations of three different LSMs and downscaled them with a CPM over the Colorado Front Range. They found considerable spreads in the climate change signals of extreme precipitation between the LSMs and in the CPM climate simulations. Even though the LSM projections show an overall decrease in the intensity of extreme events, localized maxima in the CPM climate simulations can stay as strong or even increase.

Focusing on the same region *Mahoney et al.* [2012] (see Table 1, domain b) used CPM simulations to investigate the future changes of hail storms in Colorado. They included hail as an additional hydrometeor in their CPM simulations and found a near elimination of hail at the surface although the storms intensify and more hail is generated within the cloud. The primary reason is an increase in the environmental melting level due to climate warming that results in melting the hail before it reaches the surface. This can have impacts on hail damage and flood risk in future climate.

*Rasmussen et al.* [2014] (see Table 1, domain a) used an 8 yearlong CPM climate simulations to investigate changes in the water balance of the headwaters of the Colorado River. Although they did not compare their climate change signals simulated by CPMs versus LSM results, they showed that the convection‐permitting model (CPM) climate simulations were able to capture the observed snowpack and summertime precipitation correctly in the historical simulations (see section 6.2). They further showed that even though precipitation increases in the study region in the future, runoff decreases due to increased evapotranspiration. Furthermore, consistent with previous studies by *Leung et al.* [2004], the fraction of snowfall to precipitation decreases in the future. Snowpack accumulates until January, but afterward, warming leads to snowpack reduction below 3000 m from January to March, although snowpack continues to increase above 3000 m. From April to July, snowpack decreases at all elevations in the future.

### 7.3 Changes in Tropical Cyclones

The skill in simulating tropical cyclones depends on the horizontal grid spacing of climate models (see section 6.6). There is consensus that future cyclone intensity is likely to increase (maximum wind speed and rainfall) due to warmer sea surface temperatures [*Emanuel*, 1986] whereas changes in the cyclone frequency are less certain [*Knutson et al.*, 2008; *Vecchi et al.*, 2008; *Emanuel*, 2013; *Emanuel et al.*, 2008; *Wehner et al.*, 2014]. Investigating scenarios performed with models with horizontal grid spacing (Δ*x*) between 60 km and 20 km results shows an increase in the number of intense cyclones [e.g., *Bengtsson et al.*, 2007; *Knutson et al.*, 2008]. Even though state‐of‐the‐art GCMs with horizontal grid spacing (Δ*x*) around 20 km can simulate tropical cyclone with central pressures below 900 hPa [*Murakami et al.*, 2012], investigation of changes in the cyclones structure requires CPM climate simulations that can resolve eyewall and vortex dynamics [*Braun and Tao*, 2000; *Gentry and Lackmann*, 2010].

*Kanada et al.* [2013] (see Table 1, domain n) investigated changes in the structure of extreme intense tropical cyclones in the northwestern Pacific Ocean by downscaling an ensemble of six cyclones from a GCM simulation with a CPM for future and present climate. They found that the mean central pressure minimum decreases by 23% and the maximum 10 m wind speed increases by 10%. The changes in the structure of the cyclones are significant within a radius of 50 km around the inner core. In comparison, the central pressure minimum decreases only by 5% in the GCM driving data which leads *Kanada et al.* [2013] to the conclusion that CPM climate simulations are needed to investigate the changes in extreme intense tropical cyclone.

## 8 Summary and Discussion

In this review, we present the rationale and quality of CPM climate simulations for modeling current climate, their added value and common differences compared to LSM simulations, and differences in their future climate projections compared to LSM. We briefly discuss four different modeling approaches that can be used to generate CPM climate simulations but focus on the most frequently applied method that uses grid telescoping with regional climate models (RCMs).

### 8.1 Model Setups Used for CPM Climate Simulations

The horizontal grid spacing (Δ*x*) for which numerical weather and climate models start to permit deep convection sufficiently to avoid the use of error‐prone deep convection parameterization schemes is around 4 km. The physical justification for the application of convection parameterizations starts to break down for horizontal grid spacing (Δ*x*) approximately smaller than 10 km such that parameterizations have to be either reformulated or switched off. Simulations with horizontal grid spacing (Δ*x*) in between the convection‐permitting (Δ*x* < 4 km) and the convection‐parameterized scale (Δ*x*≥10 km), called “gray zone,” should be avoided before suitable scale‐aware parameterizations are designed. Several studies report minor sensitivities for grid spacings smaller then 4 km, especially when compared to the sensitivities stemming from physical parameterizations such as microphysics. A more stringent upper bound on the horizontal grid spacing of CPM climate simulations will have to be enforced if small‐scale topographic features or land surface heterogeneities need to be resolved.

To reach such fine grid spacings, typically one to three nesting steps are necessary depending on the spatial scales of the driving global model. The parent‐grid ratio (the integer parent‐to‐nest ratios of the horizontal grid spacing) should be kept larger than 1:12 but also smaller ratios have already been applied with success.

Several approximations and simplifications in the numerics of LSMs lose their validity or cause instabilities when convection‐permitting grid spacings are approached. (1) CPM simulations demand a nonhydrostatic formulation of the dynamical core since the hydrostatic approximation is no longer valid for Δ*x* < 10 km. (2) CPM simulations demand higher accuracy and stability of numerical discretization schemes because of steeper slopes (better resolved orography). (3) The effective resolution, which is largely set by the implicit diffusion of discretizations, needs to be high in order to prevent too strong smoothing of the small‐scale dynamics and in order to not unnecessarily waste computational resources.

The setup of CPM climate simulations is often adapted from numerical weather prediction models because of the high computational costs of testing the physical settings in CPM climate simulations. Whether these settings are appropriate for simulations on climate time scales is, however, largely unknown.

### 8.2 Parameterization of Subgrid‐Scale Physics Used in CPM Simulations

Since in CPM climate simulations deep convection is explicitly modeled on the numerical grid, the initiation of convection as well as the evolution and morphology of individual clouds becomes overall more dependent on parameterizations of microphysical processes. The introduction of additional hydrometeor species, such as graupel and hail, or the simulation of the number concentration of cloud particles using two‐moment microphysics schemes can be beneficial for the representation of wintertime orographic precipitation and high cloud cover. However, several physical processes in convective and mixed‐phase clouds and their interaction with aerosols are not well understood and somewhat constrain the full potential of two‐moment schemes. Sensitivities related to cloud microphysical parameterizations and the related sensitivity of the cloud‐radiative feedback remain considerably larger than those due to mesh refinement at kilometer scales.

Since soil‐atmosphere interactions are important for many atmospheric processes, from energy fluxes at the surface that interact with near‐surface fields (e.g., 2 m temperature or humidity) to the boundary layer dynamics (including the initiation of convection), the introduction of more advanced representations of soil and vegetation processes seems to be crucial in CPM climate simulations.

### 8.3 The Added Value of CPM Climate Simulations

There is clear evidence that CPM climate simulations are able to add value to LSMs. However, it is also important to mention that CPM climate simulations are not the cure for all model biases. The largest added value can be found on small spatial and temporal scales (<100 km and subdaily), in regions with steep orography, and higher‐order statistics (e.g., extreme values). High potential for added value lies in process‐based analyses such as analysis of storm dynamics, local wind systems, and the interaction of atmospheric flows with orography (see *Houze*, [2012], for a review). Averaging over large areas and over long (e.g., climatological) time scales tends to smooth out small‐scale features and therefore also the added value of CPM climate simulations. One reason for the missing added value on large scales might be that physical parameterizations applied in LSMs are tuned to accurately represent large‐scale features. Another possibility is that the small domain sizes typically used for CPM climate simulations inhibit the modification of large‐scale features from small‐scale processes. In other words, the domain average convergence, implied by the lateral boundary conditions, strongly determines domain average properties such as precipitation [e.g., *Romps*, 2014; *Edman and Romps*, 2014].

The following added values have been found in CPM climate simulations: (1) representation of extreme precipitation on hourly time scales; (2) timing (onset and peak) of the diurnal cycle of summertime convection; (3) improved structures of (smaller and more peaked objects) precipitation objects (the smaller precipitation objects in combination with the improved diurnal cycle); (4) improved simulation of wet‐day frequencies; (5) the added value for precipitation is smaller for winter than for summer except in mountainous regions because of the stronger orographic forcing and reduced role of convection; (6) improved simulations of the buildup and melting of snowpack; (7) improvements in temperature at a height of 2 m related to improved representation of orography; and (8) simulation of the center pressures and small‐scale processes in tropical cyclones.

Due to the high computational costs, only a small number of groups investigated climate change issues using CPM climate simulations. All these studies show highly relevant differences in the regional to local climate change signals between CPM climate simulations and LSM simulations. In addition, these studies delivered insights into changes of some meteorological processes which would not have been gained with LSM. Those differences are as follows: (1) a significant increase of short‐duration extreme precipitation events during summer; (2) hail storms over mountains are likely to produce more hail in the upper parts of clouds in a warmer climate, but the amount of hail reaching the surface is likely reduced to zero due to an average increase of the melting level by 500 m; (3) stronger decrease of central pressures and stronger maximum 10 m wind speed in extreme intense tropical cyclones in the northwestern Pacific Ocean; (4) decreases in the future runoff from the Colorado Headwaters due to increased evapotranspiration which counteracts the predicted increase in runoff due to increased precipitation; (5) decreased snowfall to precipitation fraction reduces the snowpack especially in lower elevated areas and late spring; and (6) stronger increase of the daily minimum/maximum temperature in mountain/valley areas of Western Germany and parts of Luxemburg.

Climate‐relevant feedback mechanisms not only reveal different magnitudes but some even differ in sign depending on whether a LSM or a CPM was used: (1) in LSMs the soil moisture‐precipitation feedback depends on the used convection parameterization, while CPMs are able to simulate this feedback more realistically; (2) a potential increase in future forest cover, as a result from converting agricultural land to bioenergy plantations, could cause a vegetation‐atmosphere interaction which cools down regional maximum temperature at a height of 2 m.

The inability of LSMs to replicate climate‐relevant feedback mechanisms casts doubt on the reliability of regional‐scale information from LSM projections of future climates.

### 8.4 Potential for Impact Modeling

Simulations with LSMs typically have to be statistically downscaled to generate information relevant for impact models (e.g., hydrological models, glacier models, and risk models). Because of the fine grid spacing of CPM climate simulations, this statistical postprocessing can be avoided as shown in a study by *Mölg and Kaser* [2011]. A direct coupling of CPM climate simulations enables to study the influence of, e.g., thermodynamic, dynamic, hydrologic, and microphysics processes on impact‐relevant quantities such as river runoff, energy production, or risk assessment and allows for feedbacks from the impact model to the CPM. This has the potential to enhance our understanding of physical interactions between large‐scale atmospheric features and their modulation due to smaller‐scale feedbacks.

## 9 Challenges and Outlook

Although CPM climate simulations already have proven to add value to LSM simulations and to provide better insights in regional processes that are highly relevant for society and policy makers, there are several challenges which have to be addressed to exploit their full potential.

1. Turbulent parameterizations have to be developed to accurately represent the planetary boundary layer, shallow convection, subgrid‐scale cloud cover, and turbulent fluxes related to deep convective systems at kilometer scales. Recent developments, such as those by *Soares et al.* [2004], *Bogenschutz and Krueger* [2013], *Moeng et al.* [2010], and *Moeng* [2014], show promising results, but intensive evaluation is required before they can be applied in CPMs.
2. Microphysics schemes applicable in CPMs are able to simulate additional hydrometeors such as graupel or hail. While this can be beneficial for the simulation of precipitation, their interaction with radiation is often neglected because of the poorly understood optical properties of those hydrometeors.
3. Two‐moment microphysics schemes allow for a more realistic distribution of hydrometeors. However, those schemes are highly tunable, and many key processes such as drop‐drop interactions, the formation of ice particles, or the shape of the particle size distributions are not well known. To unfold the full potential of these schemes, further research is necessary to improve the representation of these processes.
4. There is very limited knowledge about cloud‐aerosol interactions with far‐reaching consequences on the development of deep convection, cloud cover, and precipitation. Cloud‐aerosol interactions are a major source of uncertainties in future climate projections. Besides coupling CPM climate simulations with aerosol modules that are able to describe particle sizes, chemical compositions, and mixing states, basic research on cloud‐aerosol interactions is needed.
5. CPM climate simulations demand for high accuracy and stability of the numerical solver to avoid instabilities and numerical diffusion. Numerical diffusion leads to effective model resolutions that are many times greater than horizontal grid spacing (Δ*x*). Reducing the numerical diffusion can therefore save computational resources. Numerical schemes with higher order of accuracy like those suggested by *Morinishi et al.* [1998] and tested by *Ogaja and Will* [2014] have high potentials to improve the model accuracy and efficiency.
6. The future application of CPM climate simulations is tightly related to developments in high‐performance scientific computing. A development especially relevant for CPM climate simulations is heterogeneous high‐performance computing architectures that combine multicore CPUs with accelerators (e.g., GPUs). An efficient performance on such architectures demands a restructuring or rewriting of parts of the model code. This may lead to speedups of up to 7X for dedicated parts of the code [e.g., *Lapillonne and Fuhrer*, 2014].
7. Not only the computational time but also the data amount processed during CPM climate simulations is challenging. Data input/output operations, handling and transfer, analysis as well as storage, and archival of such data volumes become a grand challenge [*Overpeck et al.*, 2011]. A possible solution would be to perform data analysis and visualization online during runtime thereby also making use of the parallel resources available [*Zhang et al.*, 2012; *Childs et al.*, 2013].
8. A frequently limiting factor for the detection of added value and the evaluation of CPM climate simulations is the availability of suitable observational data sets. Since the potential added value is expected at small temporal and spatial scales and for extremes, fine‐gridded observational data sets in high temporal resolution are needed. In addition, such measurements are required to cover long temporal and spatial scales in order to be utilized in climate model evaluations.
9. Detecting added value in CPM climate simulations demands suitable evaluation methods. Traditional statistics used in climate research such as climatological mean values, or annual to decadal variabilities are mostly not suitable. The new methods should be able to decompose the spectrum of variability in order to isolate small‐scale features, to analyze the tails of climatological distributions, the extreme value statistics, or to investigate joint distributions (e.g., precipitation and temperature). Furthermore, process‐based analysis methods can reveal deeper insights into the more physically and dynamically consistent atmospheric phenomena in CPM climate simulations.
10. Most of the studies presented in this review are based on results derived from a single model and/or considerably short simulation periods. Therefore, assessing the reliability and uncertainty in the presented results is challenging. More decadal‐scale CPM climate simulations are needed to improve our understanding of the climate system and involved feedbacks. A joint effort to address both added value and climate change signals in CPM climate simulations in an organized and coordinated way comparable to other programs like the coupled model intercomparison project [*Meehl et al.*, 2000] or the Coordinated Regional Downscaling Experiment (CORDEX) [*Giorgi et al.*, 2009] would be highly beneficial to establish more robust and societal and political relevant information and to support model development.
